# Mechanism of Ultrafast Intersystem Crossing in 2‐Nitronaphthalene

**DOI:** 10.1002/chem.201705854

**Published:** 2018-03-08

**Authors:** J. Patrick Zobel, Juan J. Nogueira, Leticia González

**Affiliations:** ^1^ Institute of Theoretical Chemistry, Faculty of Chemistry University of Vienna Währinger Straße 17 1090 Vienna Austria

**Keywords:** density functional calculations, intersystem crossing, 2-nitronaphthalene, non-adiabatic dynamics, photophysics

## Abstract

Nitronaphthalene derivatives efficiently populate their electronically excited triplet states upon photoexcitation through ultrafast intersystem crossing (ISC). Despite having been studied extensively by time‐resolved spectroscopy, the reasons behind their ultrafast ISC remain unknown. Herein, we present the first ab initio nonadiabatic molecular dynamics study of a nitronaphthalene derivative, 2‐nitronaphthalene, including singlet and triplet states. We find that there are two distinct ISC reaction pathways involving different electronic states at distinct nuclear configurations. The high ISC efficiency is explained by the very small electronic and nuclear alterations that the chromophore needs to undergo during the singlet–triplet transition in the dominating ISC pathway after initial dynamics in the singlet manifold. The insights gained in this work are expected to shed new light on the photochemistry of other nitro polycyclic aromatic hydrocarbons that exhibit ultrafast intersystem crossing.

## Introduction

The rate of intersystem crossing (ISC), that is, the non‐radiative transfer between electronic states of different spin multiplicity, is directly related to the size of the spin‐orbit coupling (SOC) between the states involved in the transition. SOCs, in turn, scale with the nuclear charge *Z* of the constituting atoms.^1^ Thus, one of the paradigms of traditional photochemistry has been that ISC can proceed very fast in molecules containing heavy atoms (large *Z*), for example, metal complexes, in which ISC is frequently found to occur on a femtosecond time scale.^2^ In contrast, ISC was expected to be considerably slower when the SOCs are very small, as is the case in organic molecules composed solely of light atoms of the first period. For certain classes of organic molecules, such as nitro polycyclic aromatic hydrocarbons (NPAHs)^3^, ^4^, ^5^, ^6^, ^7^, ^8^, ^9^, ^10^, ^11^, ^12^, ^13^, ^14^ and the closely related nitrobenzene derivatives,^15^, ^16^, ^17^, ^18^, ^19^, ^20^, ^21^, ^22^, ^23^, ^24^, ^25^ ISC has been measured to occur in an ultrafast sub‐picosecond timescale, challenging this paradigm.

Recently, NPAHs have gained great interest as they are widespread environmental pollutants with phototoxic effects, for example, promoting the formation of skin cancer.^26^ Among NPAHs, nitronaphthalene (NN) derivatives largely contribute to the overall ambient air toxicity.^27^ A key feature in their mode of action is the efficient population of triplet states through ISC. For example, the first report on ultrafast ISC in NN derivatives based on femtosecond fluorescence up‐conversion experiments^5^ and early semiempirical calculations^28^ for 1‐nitronaphthalene (1NN) suggested ISC in less than 100 fs. However, for other NPAH derivatives investigated in the same study^5^ the fast sub‐100 fs decay time was attributed to a conformational relaxation in the initially populated excited state involving the re‐orientation of the nitro group. Later studies on 1NN using different solvents^6^ and sub‐ps‐resolved transient absorption spectroscopy experiments^7^ reassured that the decay of S_1_ occurs within 100 fs and it was established that relaxation within the triplet manifold (T_*n*_–T_1_) proceeded on a time scale of 1–16 ps.

Further transient absorption spectroscopy experiments on 1NN, as well as on 2‐nitronaphthalene (2NN), and 2‐methyl‐1‐nitronaphthalene (2M1NN)^3^, ^4^, ^29^ showed that after excitation to the lowest‐energy absorption band, all three NN derivatives exhibit multiexponential decay signals with similar lifetimes in the order of *τ*_1_=0.1–0.4 ps, *τ*_2_=1–3 ps, and *τ*_3_=6–10 ps, that is, separated by roughly one order of magnitude.For 2NN, *τ*_1_ and *τ*_2_ were assigned to ISC (S_1_→T_n_) and internal conversion (IC) within the triplet manifold (T_n_→T_1_), respectively, while *τ*_3_ was attributed to vibrational cooling in the hot T_1_ state. The assignment of lifetimes for 1NN and 2M1NN was similar, with the exception that, since 1NN and 2M1NN exhibit photodegradation upon UV irradiation, *τ*_1_ was supposed to describe a bifurcation including both, ultrafast ISC to the triplet states and ultrafast conformational relaxation to a singlet state with dissociative character. It was assumed that the difference in the excited‐state dynamics between 2NN and 1NN/2M1NN is controlled by the nitro group torsion, that is, while the small torsion in the nearly planar 2NN drives this system only towards ISC after photoexcitation, the larger angles in 1NN/2M1NN allow the system to populate more efficiently the dissociative singlet state, characterized by a close‐to‐perpendicular torsion angle. The femtosecond ISC was justified by the presence of large SOCs—calculated as approximately 65 cm^−1^ for 1NN, which was considered large for purely organic molecules.^30^ However, reaction rates calculated in ref. 30 using these SOCs and Fermi's golden rule predicted ISC to occur only on a ps time scale (*k*_ISC_=1.8–5.2×10^11^ s^−1^, i.e., 1.9–5.2 ps).^30^

Besides experimental studies, a small number of studies calculated excited states and potential‐energy surfaces along selected nuclear degrees of freedom to explain the photophysics of nitroaromatic compounds.^30^, ^31^, ^32^, ^33^ However, dynamics simulations on nitroaromatic compounds have not been carried out so far.

Summarizing the previous work, it appears that different timescales for ISC in NN derivatives have been postulated until now: sub‐100 fs,^5^ within 100–400 fs,^3^, ^4^ or one order of magnitude slower.^30^ The present work has been conceived to find theoretically the timescales governing the deactivation of NN derivatives upon photoexcitation and thus revealing the reasons responsible for the ISC and its timescale. This is the first excited‐state dynamics study on a NN derivative using ab initio nonadiabatic molecular dynamics including singlet and triplet states on the same footing. The system 2NN has been chosen as a prototype, as this molecule seems to be the most efficient in undergoing ISC without the presence of dissociative photodegration competing pathways, as in 1NN or 2M1NN.^3^, ^4^, ^29^ Our results demonstrate that ISC happens with a characteristic time of approximaely 0.7 ps supporting a mechanism, that, while not on a (sub‐) 100 fs time scale, is indeed ultrafast for an organic chromophore. Most importantly, we show that both the electronic structure and the nuclear configuration of 2NN in the precursor singlet excited state are not significantly altered on the way to populate the triplet states, favouring efficient ISC.

## Results and Discussion

### Deactivation mechanism

**Figure 1:**
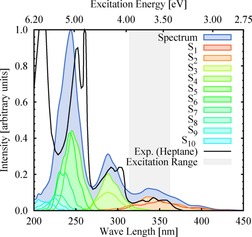
Calculated absorption spectrum of 2NN in the gas phase, individual contributions of the singlet states to the spectrum, experimental reference spectrum in *n*‐heptane,^63^ and excitation energy range for setting‐up the dynamics simulations.

**Figure 2:**
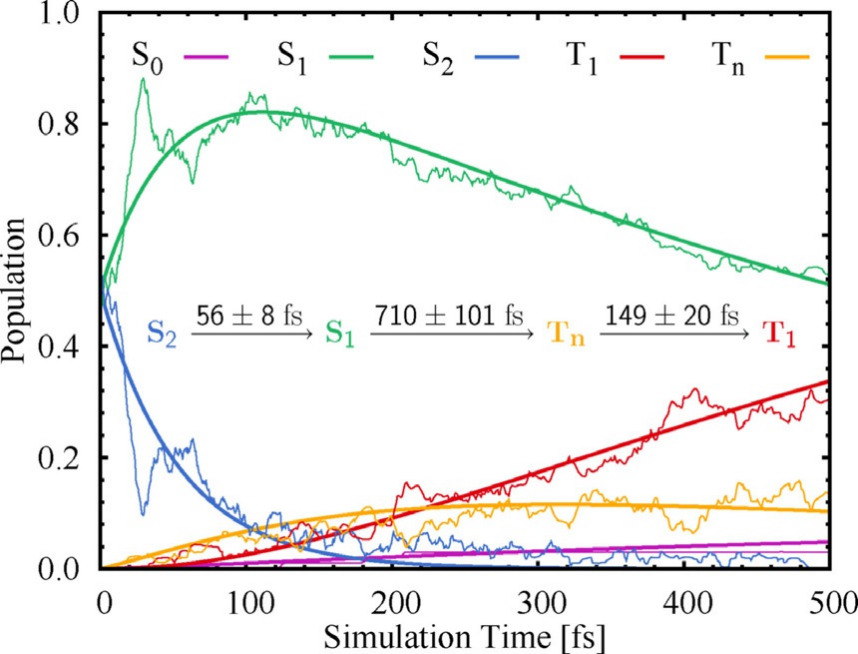
Time evolution of the spin‐orbit free state populations (thin lines) in the first 500 fs of the excited‐state dynamics of 2NN. The populations of the excited triplet states *T_n_* (*n*=2–6) have been combined into one. See Figure S5 in the Supporting Information for the individual contributions of all triplet states. Thick lines represent fits based on the first‐order processes given in Equation (1).

As in the experiments, we started our simulations by exciting to states around the maximum of the lowest‐energy UV absorption band (see Figure 1), which corresponds to the states S_1_ and S_2_. After excitation we follow the time evolution of the spin‐orbit free electronic‐state populations as shown in Figure 2 along the dynamics. The S_2_ population readily decreases until nearly zero within 200 fs, whereas the S_1_ population increases reaching a maximum after approximately 100 fs before it steadily decreases for the remaining simulation time. The triplet states are populated already after few tens of femtoseconds. Notably, whereas the population of the higher‐lying triplet states T_*n*_ (*n*=2–6) becomes steady after approximately 200 fs, the population of the T_1_ continues growing. At the end of the simulation time, *t*=500 fs, the population of the triplet states has reached approximately 45 %. During this time, approximately 3 % of the population has been transferred back to the electronic ground state S_0_.

To derive a mechanism for the excited‐state deactivation and identify the major reaction channels, we first examined the net amount of hops between different states (Table S2 in the Supporting Information). We found that the initial population of the S_2_ state is transferred mainly to the S_1_ state, from which part of the trajectories undergo ISC to triplet states T_*n*_ (*n*=2–6), before they relax by IC within the triplet manifold to the T_1_ state. Accordingly, the first 500 fs of the excited‐state dynamics of 2NN upon UV photoexcitation are governed by the simple kinetic model:

(1) $$S{}_{2}\overset{k{}_{S}}{\underset{}{\to }}S{}_{1}\overset{k{}_{\mathrm{ISC}}}{\underset{}{\to }}T{}_{n}\overset{k{}_{T}}{\underset{}{\to }}T{}_{1}$$

Fit functions for the state populations as well as time constants *τ*_i_=1/k_*i*_ based on this mechanism are also shown in Figure 2. Error margins for the time constants were calculated using the bootstrap method.^34^ Details on the fitting and bootstrapping calculations as well as discussion of minor reaction channels can be found in Sections S2.1–S2.2 of the Supporting Information.

For the first step, that is, IC from the S_2_ to the S_1_, a time constant of *τ*_S_=56± 8 fs is obtained. From the S_1_, the system undergoes ISC to the triplet manifold with a time constant of *τ*_ISC_=0.7 ps before it relaxes to the T_1_ within approximately 150 fs (*τ*_T_). Comparing these time constants to experimental results, we find that our *τ*_S_ and *τ*_ISC_ can be attributed to the time constants obtained for the two initial processes in the transient absorption spectroscopy experiments, that is, *τ*_1_=0.11± 0.05 and *τ*_2_=2.1± 0.1 ps for 2NN in cyclohexane.^3^ However, whereas in our simulations *τ*_S_ and *τ*_ISC_ belong to the S_2_→S_1_ IC and the S_1_→T_*n*_ ISC reactions, respectively, *τ*_1_ and *τ*_2_ were assigned experimentally to ISC and relaxation dynamics within the triplet manifold, respectively. Certainly, we are aware that there is a large difference between the experimental time constant *τ*_2_ and our predicted *τ*_ISC_. However, for our discussion of the excited‐state mechanism of 2NN, we consider it is sufficient that both time constants are of the same magnitude. Note that our model only analyzes the first 500 fs of the excited‐state dynamics assuming simple first‐order kinetics. Based on this model, it is, nevertheless, possible to estimate longer time constants (e.g., *τ*_ISC_) as well as time constants of processes only commencing (e.g., *τ*_T_) within our simulation window, that is, we do not have to wait until ISC to the triplets is completed to observe IC within the triplets.

The third time constant obtained experimentally (*τ*_3_=6−10 ps) is too large to be reproduced by our simulations and, therefore, is left out of our discussion. What is important to realize is that our *τ*_T_=150 fs has not been resolved experimentally as it is much smaller than the time constant of the preceding reaction (*τ*_2_). Accordingly, we propose that the experimental^3^, ^4^ *τ*_2_ is an effective time constant involving two processes, ISC to and IC within the triplet states. As an exercise, it is possible to set a theoretical model in which population goes directly from S_2_ to an intermediate species and then to T_1_; the calculated effective time constant in this case is 917 fs, which is larger than *τ*_ISC_ alone, in line with the experimentally^3^, ^4^ measured *τ*_2_.

Notice that in our mechanism, *τ*_T_ predicts IC within the triplet states (150 fs) on a similar timescale as IC within the singlet states (60 fs). The two‐fold difference can be easily attributed to the fact that IC in the singlets involves only deactivation from S_2_ to the S_1_, whereas in the triplet manifold the deactivation requires a consecutive nonradiative decay through multiple triplet states (see Table S2, Supporting Information), consequently, being slower. The present interpretation is different from that proposed previously,^3^, ^4^ that is, that IC within the triplets is an order of magnitude slower than it is in the singlets.

To disentangle the further details of the proposed mechanism and contrast it with the experimental findings, hereafter we will target the following three key issues: 1) the nature of the initially excited states, 2) the actual ISC process, and 3) the dynamics occurring in the singlet manifold.

### Initial excited states

In the transient absorption spectroscopy experiments, the system is excited to the first absorption band. At the optimized, minimum‐energy Franck‐Condon (FC) geometry the lowest‐energy bright state is the S_1_ state, the intramolecular charge‐transfer ππ* state (S_CT_(ππ*)), and thus the experimental model assumes that after excitation only the S_1_ state is populated. Obviously, from S_1_ the system can only undergo ISC—explaining why the assignment of *τ*_1_ was attributed to ISC.

**Figure 3:**
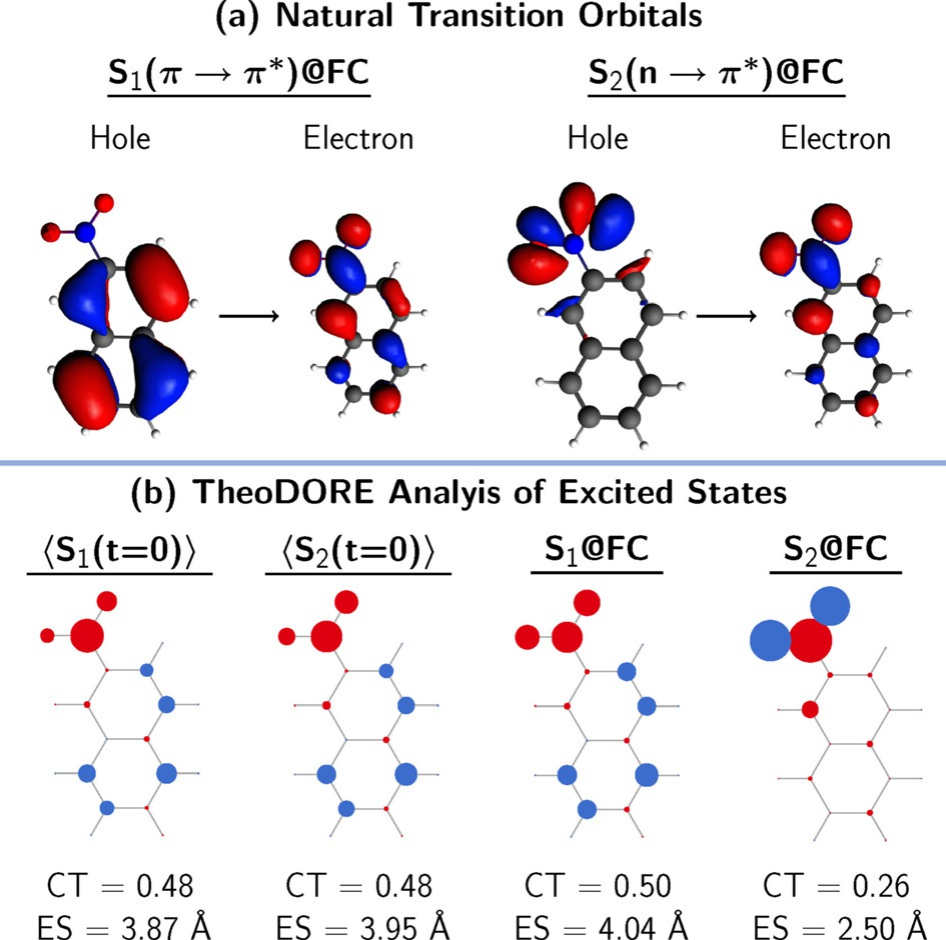
a) Natural transition orbitals describing the S_1_ and S_2_ states at the FC geometry. b) Atomic electron/hole difference populations (red/blue circles), charge transfer numbers (CT), and exciton sizes (ES) of the states initially populated in the simulation (⟨S_1,2_(*t*=0)⟩) and the S_1_ and S_2_ states at the FC geometry (S_1,2_@FC). In the excited states, electron density is transferred from the blue circles (holes) to the red circles (electrons).

However, the inclusion of vibrational motion, as available from the zero‐point energy and thermal energy, results that the bright state is not only composed of the S_1_ state, but also of the S_2_ (recall Figure 1). This is because vibrational motion brings the molecule out of plane, the orbitals mix, and the charge‐transfer S_CT_(ππ*) state, which is the S_1_ state at the optimized FC geometry, can be either the S_1_ or S_2_ state when vibrational sampling is introduced. This is best illustrated by examining the character of the S_1_ and S_2_ states computed at the FC geometry and that of all 99 initial states at *t*=0 fs (⟨S_1,2_(*t*=0)⟩), by a TheoDORE analysis of the transition density matrix.^35^, ^36^, ^37^, ^38^ Figure 3 depicts the natural transition orbitals of the states S_1_–S_2_ at the FC geometry (panel a) with the atomic electron/hole difference populations, together with the charge‐transfer numbers (CT), and exciton sizes (ES) of the ensemble of the initial states (⟨S_1,2_(*t*=0)⟩) compared with those at the S_1_ and the S_2_ at the FC geometry (panel b). As can be seen, the electron/hole difference populations, CT, and ES are the same for initial states S_1_(*t*=0)/S_2_(*t*=0) and the S_1_(ππ*)@FC state. Thus, as in experiment, also in the simulations it is only the bright S_CT_(ππ*) that is populated initially. However, this state corresponds to the S_1_ and the S_2_ states at different geometries in the vibrational ensemble. Specifically, for our simulations employing a 0.5 eV broad energy range around the absorption band maximum, this corresponds to 49 and 50 stochastic initial conditions in the S_1_ and S_2_ states, respectively, from which the nonadiabatic simulations are started. Accordingly, one can expect that there are initial relaxation dynamics within the singlet states, and this process is characterized by the time constant *τ*_S_.

### Electronic structure in intersystem crossing pathways

**Figure 4:**
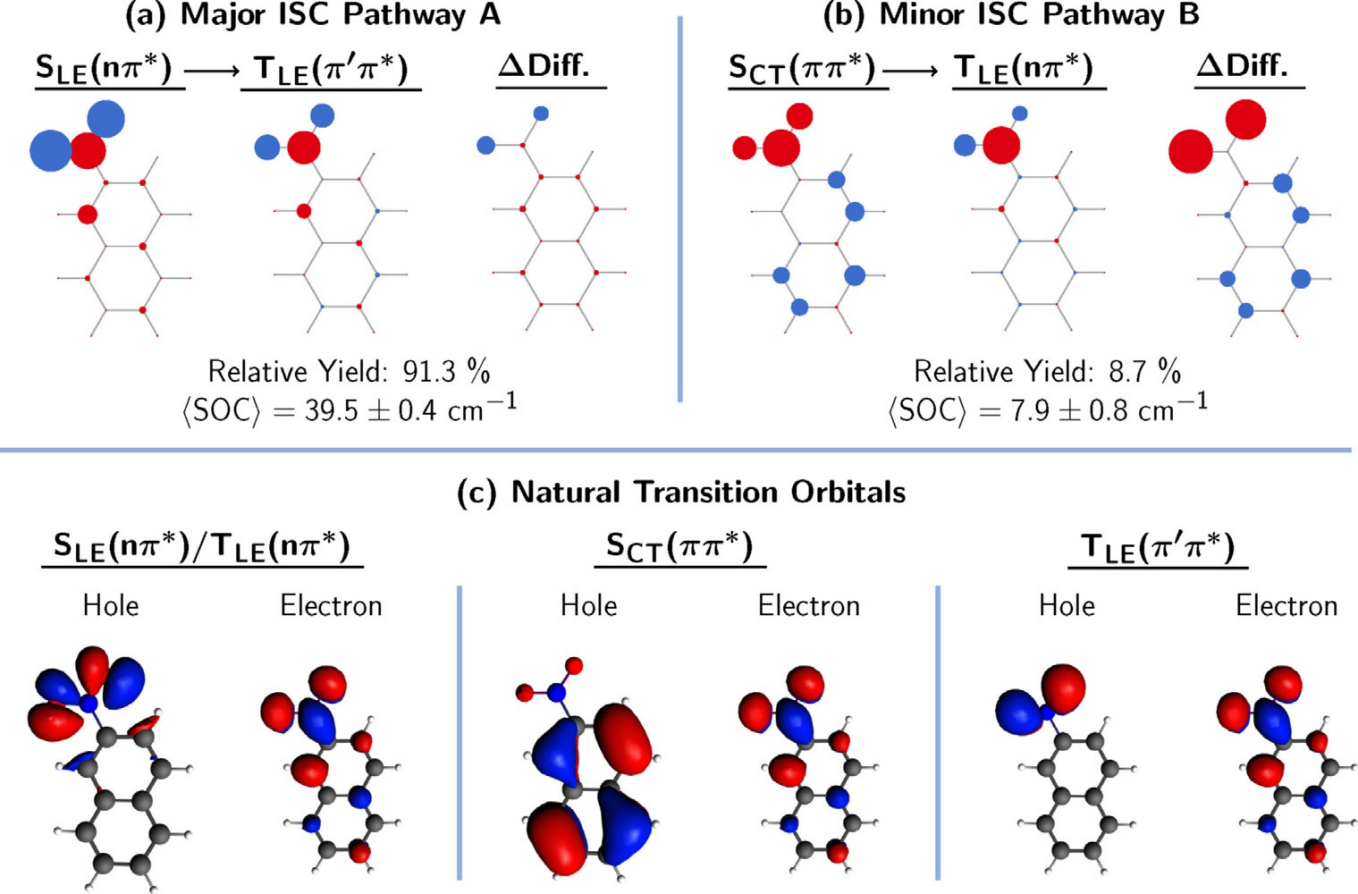
a/b) Atomic electron/hole (red/blue circles) difference populations for the singlet and triplet states involved in the major/minor ISC pathways, relative contributions of both pathways after 500 fs, and average spin‐orbit coupling (SOC) matrix elements between the respective singlet and triplet states. ΔDiff is the difference between the electron/hole difference populations of the respective singlet‐triplet state pair. c) Natural transition orbitals describing the excited states involved in the ISC pathways—for illustration purposes only (read section S2.3 in the Supporting Information).

Next, we shall examine the dynamics within the singlet manifold and through the ISC process. First, we elucidate the nature of the ISC process by examining all ISC hopping events in the trajectories. Interestingly, we find two different pathways when analyzing the singlet and triplet excited states at the hopping points. The majority of ISC hops occur from a locally excited singlet *n*π* state [S_LE_(*n*π*)] to a locally excited triplet π′π* state [T_LE_(π′π*)] (pathway A), while a minor fraction of ISC hops occur from a charge‐transfer singlet ππ* state [S_CT_(ππ*)] to a locally excited triplet *n*π* state [T_LE_(*n*π*)] (pathway B). Figure 4 shows the average atomic electron/hole difference populations of the singlet and triplet states involved in these two pathways and the natural transition orbitals that describe these states.

Interestingly, the electron/hole difference populations of the donor singlet and acceptor triplet states in the major pathway are very similar, whereas they differ considerably for the donor–acceptor pair in the minor pathway, as shown in Figure 4 a/b. Thus, the electronic distribution requires only very small alterations in the major pathway while it changes considerably in the minor pathway. This is best shown by the difference between the atomic electron/hole difference populations of the respective singlet–triplet state pair (ΔDiff in Figure 4), that is, ΔDiff directly shows the atomic contributions of the electron flow required for the hop.

The different extent of the electron flow in the major and minor ISC pathways can also be mapped by examining the natural transition orbitals describing the excited states in the ISC transitions at the hopping geometries, see Figure 4 c. For the S_LE_(*n*π*)→T_LE_(π’π*) transition of the major pathway we find that the electron orbital, π*, is virtually the same for both donor (singlet) and acceptor (triplet) state and the hole orbitals *n* and π′—both located at the nitro group—also share strong resemblance. In the simple orbital picture, the S_LE_(*n*π*)→T_LE_(π’π*) transition, thus, corresponds to transferring an electron from the π′ to the *n* orbital. Both orbitals are mainly antisymmetric linear combinations of atomic *p*‐orbitals located at the oxygen atoms of the nitro group, lying either on the molecular plane (p(x,y)O1 −p(x,y)O2 →*n*) or perpendicular to it (p(z)O1 −p(z)O2 →*n*). The π′→*n* electron transfer is realized simply by changing the angular momentum of the electron in the p orbitals of the oxygen atoms, going from p^(*z*)^ to p^(*x*,*y*)^—following textbook El‐Sayed rules.^39^ For the hopping geometries in the major pathway, the SOC matrix elements amount to approximately 40 cm^−1^ (Figure 4), which, though sizable and similar to other organic molecules for which ISC has been predicted,^40^, ^41^, ^42^ is smaller than the 65 cm^−1^ value calculated for 1NN.^30^

The favorable interaction of the electronic configurations of the singlet and triplet state—as shown by the similar electronic transition density properties, seen both in terms of the natural transition orbitals and in terms of the electron/hole populations—is likely a key factor favoring the S_LE_(*n*π*)→T_LE_(π’π*) transition as the major ISC pathway. In the following, we will identify additional reasons favoring the S_LE_(*n*π*)→T_LE_(π’π*) pathway.

### Initial dynamics in singlet manifold

The majority of ISC hops originate from the S_LE_(*n*π*) state, whereas only a small fraction of trajectories take the minor ISC pathway starting in the S_CT_(ππ*) state. Since the latter corresponds to the initially excited singlet state ⟨S_1,2_(*t*=0)⟩, the beginning of the excited‐state dynamics needs to be driven by IC from the S_CT_(ππ*)=⟨S_1,2_(*t*=0)⟩ to the S_LE_(*n*π*) state. As both states strongly differ in their electronic character, we can distinguish them by their dipole moment and monitor the IC by following the dipole moments of the trajectories *μ*(t).

**Figure 5:**
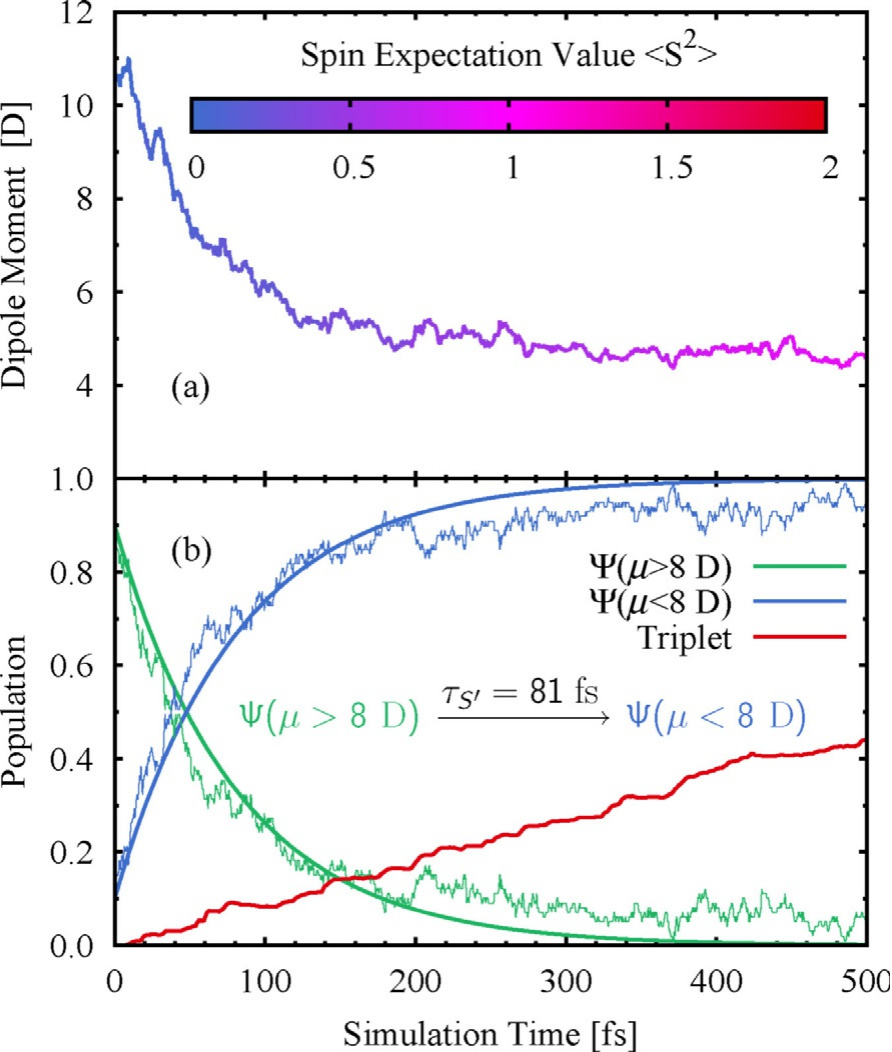
a) Mean value of the dipole moment ⟨*μ*(*t*)⟩ of all trajectories. Color of the line corresponds to the spin expectation value ⟨S^2^⟩=S(S+1). b) Time evolution of the quantum populations of all states with a dipole moment smaller/larger than 8 D and of all triplet states.

Figure 5 a shows the average dipole moment of all trajectories ⟨*μ*(t)⟩. Initially, it values 10.7± 1.1 D before it drops down to 6.0±0.7 D around 100 fs, and decreases further to 4.6± 0.5 D after 500 fs. Although triplet states are already populated after few tens of femtoseconds, the majority of the excited state population remains in the singlet manifold at the beginning of the simulation. Thus, the initial decrease of ⟨*μ*(t)⟩ is primarily due to the S_CT_(ππ*)→S_LE_(*n*π*) IC which occurs with a time constant of *τ*_S_=81 fs (see Section S2.5 in the Supporting Information). This time constant is better suited to describe the dynamics in the singlet manifold than the previously introduced one of *τ*_S_=56 fs, because *τ*_S_ describes IC involving a transition between the two spectroscopic states S_CT_(ππ*) and S_LE_(*n*π*), which is the process monitored in experiment. In contrast *τ*_S_ captures all S_2_→S_1_ processes regardless of the character of the states, that is, adiabatic transitions such as S_2_(ππ*)→S_1_(ππ*) and nonadiabatic transitions, such as S_2_(ππ*)→S_1_(*n*π*). The time constant *τ*_S’_=81 fs is an order of magnitude faster than the ISC (*τ*_ISC_≈1 ps), that is, population shifts gradually from the donor state of the minor ISC pathway to the donor state of the major ISC pathway in which population accumulates before ISC occurs. Thus, ISC through a minor pathway is quenched dynamically, favoring the major ISC pathway as time evolves.

**Figure 6:**
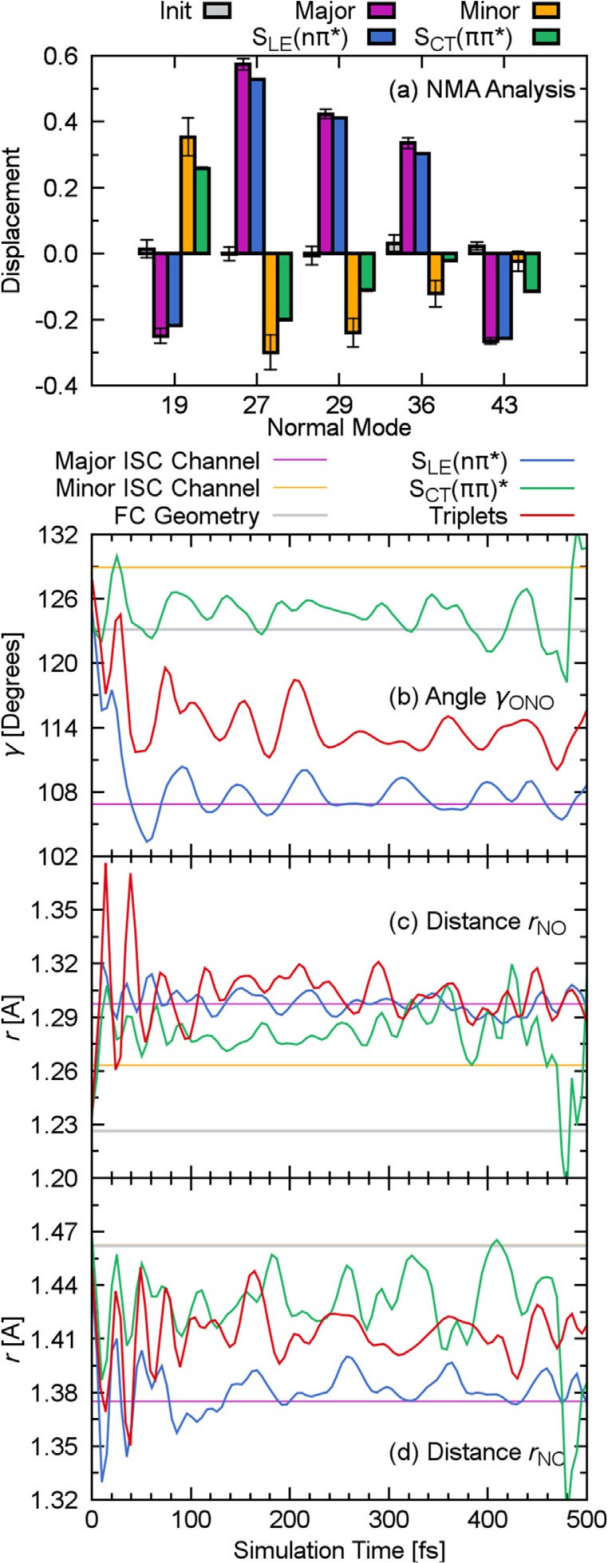
a) Normal mode analysis (NMA) of the geometries in the initial conditions, all hopping geometries in the major and minor pathway, and all geometries in the S_LE_(*n*π*) and S_CT_(ππ*) states. b–d) In dashed lines, the averaged internal coordinate at the hopping geometries of the major (violet) and minor (orange) ISC pathways, and at the FC geometry (gray). In solid lines, the time evolution of the internal coordinate for the corresponding trajectories in the S_LE_(*n*π*) (blue), S_CT_(ππ*) (green) or any triplet state (red).

As a last part of our discussion, we shall analyze the nuclear motion governing the underlying dynamics based on a normal mode analysis (NMA),^43^, ^44^ for which the nuclear motion is expressed in terms of the normal modes of the ground‐state equilibrium geometry. The NMA allows us to identify the important modes for the ISC pathways based on their large displacement at the hopping geometries (Section S2.6, Supporting Information). These are the normal modes 19, 27, 29, 36, and 43—collected in Figure 6 a ‐and for which the displacement vectors are shown in Figure S11 in the Supporting Information.

These modes show a very small displacement for the initial geometries, as expected from the harmonic Wigner distribution, but a substantial change at the ISC hopping geometries in the major and minor pathways (Figure S9, Supporting Information). This means that the system requires substantial nuclear motion to reach the potential‐energy regions at which ISC can take place. With the exception of mode 43, the displacement at the hopping geometries in the minor and major pathways is of opposite sign meaning both pathways take place at distinct, far away regions of the potential‐energy surface.

We now compare the normal‐mode displacements at the hopping geometries of the major and minor pathways with the average of their singlet donor states S_LE_(*n*π*) and S_CT_(ππ*), respectively. As can be seen in Figure 6 a, the differences between the normal‐mode displacement of the S_LE_(*n*π*) state and the hopping geometries of the major pathway are much smaller than for the S_CT_(ππ*) and the minor pathway, thus, revealing another key feature that favors the S_LE_(*n*π*)→T_LE_(ππ*) over the S_CT_(ππ*)→T_LE_(*n*π*) pathway: the system does not only spend more time in the S_LE_(*n*π*) state than in the S_CT_(ππ*), but in the S_LE_(*n*π*) state it is on average also closer to the S_LE_(*n*π*)→T_LE_(π’π*) hopping region, that is, not requiring any further large motion. In contrast, in the S_CT_(ππ*) state, the molecule needs to undergo considerable structural changes to reach the hopping region of the minor ISC pathway.

The information obtained from the NMA can also be translated into some relevant internal coordinates. Specifically, the analysis of the normal modes identified primarily three internal coordinates that change the most: the angle *γ*_ONO_ between the atoms of the nitro group, the distances *r*_NO_ between the N and the O atoms, and the distance *r*_NCα_ between the N atom and its neighboring C atom. The time evolution of the averages of *γ*_ONO_, *r*_NCα_, and *r*_NO_ is plotted in Figure 6 b–d, for the trajectories in the S_LE_(*n*π*), S_CT_(ππ*), or in the triplet states (see also Figure S11, Supporting Information). The averages of these coordinates calculated at the hopping geometries of the major and minor ISC channels and the reference value calculated at the FC geometry are also shown. In agreement with the NMA analysis, the averages of all three coordinates of trajectories in the S_LE_(*n*π*) state are for most of the simulation time very close to the averages of the hopping geometries of the major ISC channel. In contrast, the average internal coordinates of trajectories in the S_CT_(ππ*) state show a larger deviation from the averages of the hopping geometries of the minor ISC channel. This supports the hypothesis that the S_LE_(*n*π*)→T_LE_(π’π*) pathway is favored because the nuclear conformations of trajectories in the S_LE_(*n*π*) state are closer to the hopping region than in the case of the S_CT_(ππ*) state.

## Conclusions

**Figure 7:**
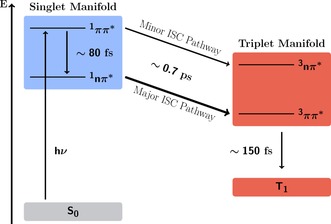
Mechanism of the excited‐state dynamics of 2NN.

The simulation of the excited‐state dynamics of 2NN allowed us to obtain a clear‐cut deactivation mechanism that is summarized in Figure 7. After photoexcitation to a S_CT_(ππ*) state, most of the excited‐state population is transferred to a S_LE_(*n*π*) state through IC with a sub‐100 fs time constant *τ*_S_. This timescale is also found experimentally,^3^, ^4^ but it was attributed to ISC. In contrast, our simulations reveal that ISC in 2NN takes place on a longer timescale (*τ*_ISC_=0.7 ps) and proceeds through two distinct reaction pathways S_LE_(*n*π*)→T_LE_(ππ*) (major pathway) and S_CT_(ππ*)→T_LE_(π’π*) (minor pathway). Only the minor ISC pathway was previously suggested in the literature.^3^, ^4^ Then, after ISC to the triplet manifold, nonradiative deactivation within the triplet states happens with a timescale of *τ*_T_=150 fs.

The very high ISC rate in 2NN is due to three key features that lead the system efficiently through the major ISC pathway. First, its singlet ISC donor state, S_LE_(*n*π*), is rapidly populated and longer‐lived than the other singlet excited states. Second, once the precursor singlet state is populated, the nuclear configurations of 2NN are very close to the configurations that allow ISC and, thus, only small geometrical arrangements are necessary. Third, at the ISC geometries, the transition from the singlet donor (S_LE_(*n*π*)) to the triplet acceptor (T_LE_(ππ*)) state requires only a small change in the electronic density, that is, only the angular momentum of one electron is required to change, wheras the remaining electronic density remains static.

The insights gained in this study thus clarify the factors responsible for the ultrafast ISC in 2NN and are expected to help unravelling the dynamics in other NPAH derivatives. Work on the excited‐state dynamics on other nitronaphthalene derivatives, including a focus on temperature effects, is in progress.

## Methods

### Non‐adiabatic simulations

To simulate the excited‐state dynamics of 2NN within the singlet and triplet manifold, we used the surface hopping including arbitrary couplings (SHARC) approach.^45^, ^46^, ^47^ SHARC is an extension of the family of trajectory surface‐hopping methods,^48^ in which the nuclei are propagated classically on quantum‐chemical potential‐energy surfaces calculated on‐the‐fly.^49^ There exist a number of further implementations of surface‐hopping, which allow including non‐adiabatic couplings and SOCs on the same footing.^50^, ^51^, ^52^, ^53^ Alternatively, it is also possible to include SOCs in accurate quantum wave packet dynamics^54^ or in the multiconfigurational time‐dependent Hartree method,^55^ including a selected number of degrees of freedom. Lying somewhere between quantum dynamics and surface hopping—both in terms of accuracy and computational costs—intersystem crossing processes can also be described with ab initio multiple spawning.^56^

In this work, the energies, gradients, non‐adiabatic couplings from wave function overlaps,^57^ and SOCs are obtained on‐the‐fly at the PBE0^58^, ^59^/DZP^60^ level of theory, as implemented in the ADF2016 program package.^61^ This level of theory is chosen as PBE0 is able to reproduce satisfactorily the experimental absorption spectrum of 2NN in methanol and acetonitrile.^62^ The calculations in this study are performed in gas phase and it is assumed that the excited‐state energies of 2NN in the gas phase calculated with PBE0 are also reliable. The solvent is excluded as previous experiments^3^, ^4^ on 2NN performed in different solvents (cyclohexane and acetonitrile) provided very similar results. Thus, as the excited‐state dynamics of 2NN does not appear to be sensitive to environment effects, we expect gas‐phase calculations to yield results comparable to the experimental data.^3^, ^4^

The initial conditions required for the nonadiabatic simulations were obtained from 1000 geometries sampled from a temperature‐dependent Wigner distribution at *T*=300 K (see below). For each geometry, the ten lowest excited states were calculated and used to simulate the absorption spectrum by convoluting the resulting stick spectra. The obtained gas‐phase spectrum (Figure 1) agrees well with the experimental absorption spectrum obtained in the nonpolar solvent *n*‐heptane.^63^ At the equilibrium geometry, the first singlet and triplet excited states S_1_/T_1_ state have intramolecular ππ* charge‐transfer character, whereas the S_2_/T_2_ states are *n*π* local excitations within the nitro group. The energy, oscillator strength, and character of these and remaining excited states is listed in Table S1 of the Supporting Information.

From the ensemble, 105 random geometries were selected and propagated for 500 fs. Their trajectories were started at the corresponding bright excited state, considering a 0.5 eV energy range around the maximum of the calculated absorption band (gray area in Figure 1). This resulted in 51 and 54 trajectories starting from the S_1_ and S_2_ states, respectively. Note that the character of the electronic states can change due to vibrational motion accounted for within the initial ensemble, and for this reason the bright state within the ensemble is for some geometries S_1_ and for others S_2_. Due to convergence problems and hops to inactive states during the simulation time, 6 trajectories had to be excluded, thus leaving 99 trajectories for the statistical analysis presented in this article (for more details, see Section S1 of the Supporting Information).

To perform and analyze the results of nonadiabatic dynamics simulations, different electronic‐state representations can be employed. Within SHARC, the individual trajectories are propagated in a basis of so‐called spin‐adiabatic electronic states, which are states that diagonalize the full Hamiltonian, that is the molecular Hamiltonian plus the SOC Hamiltonian.^47^ On this basis, the SOCs between the electronic states transform into localized couplings, so that trajectories hop only near crossing regions and the states are spin‐mixed. To analyze the full ensemble of trajectories it is nevertheless more convenient to consider spin‐orbit free states, that is, as described by the bare molecular Hamiltonian. In this representation the electronic states are only differentiated by their spin multiplicity and are ordered according to their energy within their spin manifold, that is, leading to the state labels S_0_–S_2_ and T_1_–T_6_. As these states can change character during the dynamics, this classification allows for an easier monitoring of the principle reaction pathways, for example, relaxation in the singlet manifold or the intersystem crossing between any singlet and triplet states.

### Temperature‐dependent Wigner sampling

The Wigner distribution function, which maps the classical phase space to the quantum distribution of the coordinates *q* and momenta *p*, can be written as^64^

(2) $$W\left[\Psi \right]\left(q,p\right)=\frac{1}{\left(2\pi \hbar \right){}^{N}}\int ds\;\exp\left(ip\cdot s/\hbar \right)\Psi \left(q-s/2\right)\Psi \left(q+s/2\right)\;$$

in which s is a spatial variable, *N* is the number of dimensions, and *Ψ* is the wave function of the system. W[Ψ] is a functional of the wave function. Commonly, one employs the vibrational ground‐state wave function of the harmonic oscillator *ϕ*_0_ as Ψ when using a Wigner distribution to generate initial conditions for molecular dynamics simulations. The assumption, that the system is always in the vibrational ground state, refers to the theoretical situation of zero‐temperature. To consider a system at a finite temperature ‐say, *T*=300 K ‐population of excited vibrational levels has to be allowed. The probability *P*_n_ that a vibrational state *ϕ_n_* is populated is given by

(3) $$P{}_{n}\left(T\right)=\frac{\exp(-\beta E{}_{n})}{\Sigma {}_{n}\;\;\exp\left(-E{}_{n}\right)}=\frac{\exp(-\beta E{}_{n})}{Z}\;$$

in which *β*=(*k*_B_ *T*)^−1^ and *Z* is the canonical partition function. Thus, when generating the initial conditions for a system at a finite temperature, one can use the different Wigner distribution functions W[*ϕ_n_*] for the different vibrational states *ϕ_n_* according to their temperature‐dependent population *P_n_*(*T*).

Using thermal Wigner sampling, the system possesses both the zero‐point and the thermal energy, that is, it possesses larger momenta, which can increase reaction rates, and a larger total energy, which can open up new reaction channels by giving the system more energy to overcome (small) barriers. The thermal energy is due to the population of vibrational excited states which also changes the conformational distribution, especially for low‐frequency modes such as the nitro group torsion and other out‐of‐plane torsional modes in 2NN. Thus, including the effects of a non‐zero temperature in the Wigner sampling yields initial conditions that are in closer resemblance to the conditions in the experiment than Wigner sampling at zero temperature.

## Conflict of interest

The authors declare no conflict of interest.

## Supporting information

As a service to our authors and readers, this journal provides supporting information supplied by the authors. Such materials are peer reviewed and may be re‐organized for online delivery, but are not copy‐edited or typeset. Technical support issues arising from supporting information (other than missing files) should be addressed to the authors.

Supplementary
