# Supplementary material for: Mechanism of Ultrafast Intersystem Crossing in 2‐Nitronaphthalene
Source: Chemistry. 2018 Mar 8;24(20):5379–87. doi: 10.1002/chem.201705854 (PMC5947663; doi:10.1002/chem.201705854)
Supplement: Supplementary file 1 — Supplementary [file CHEM-24-5379-s001.pdf]

CHEMISTRY
A European Journal

Supporting Information

# Mechanism of Ultrafast Intersystem Crossing in 2-Nitronaphthalene

J. Patrick Zobel, Juan J. Nogueira,* and Leticia González*[a]

chem_201705854_sm_miscellaneous_information.pdf

# The Mechanism of Ultrafast Intersystem Crossing in 2-Nitronaphthalene

J. Patrick Zobel, Juan J. Nogueira,* and Leticia González*

*Institute of Theoretical Chemistry, University of Vienna, Währinger Straße 17, A-1090 Vienna, Austria*

E-mail: nogueira.perez.juanjose@univie.ac.at; leticia.gonzalez@univie.ac.at

## Supporting Information Available

This document contains complete computational details and raw data used for the analysis presented in the main paper, as well as additional analysis and explanations, not included in the main manuscript.

# Contents


**Supporting Information Available** **S1**

**S1 Methodology** **S3**

S1.1 Computational Details . . . . . . . . . . S3

S1.2 Problems Encountered in the Simulations . . . . . . . . . . S9

**S2 Mechanism of Excited-State Dynamics** **S12**

S2.1 Major Reaction Channels . . . . . . . . . . S12

S2.2 Minor Reaction Channels . . . . . . . . . . S15

S2.3 ISC Pathways . . . . . . . . . . S15

S2.4 Spin-Orbit Couplings in the ISC Pathways . . . . . . . . . . S18

S2.5 Initial Dynamics in the Singlet States . . . . . . . . . . S20

S2.6 Normal Mode Analysis . . . . . . . . . . S22

S2.7 Nuclear Motion . . . . . . . . . . S24

**References** **S26**

# S1 Methodology

## S1.1 Computational Details

The excited-state dynamics of 2-nitronaphthalene (2NN) in gas phase were simulated using a local version of the surface hopping including arbitrary couplings (SHARC) approach.[1–3] Density functional theory (DFT) and time-dependent DFT (TDDFT) calculations were performed at the PBE0[4,5]/DZP[6] level of theory using the ADF2016 program package[7] and the recently implemented SHARC/ADF interface.[8]

**Initial Conditions.** Before simulating the excited-state dynamics, first a ground-state geometry optimization was performed and normal modes and vibrational frequencies were calculated at the minimum-energy geometry (Figure S1). Then, 1000 initial conditions were sampled around the optimized geometry using a harmonic Wigner distribution[9] for a temperature of $T = 300$ K. For each structure, the 10 lowest excited singlet and triplet states were calculated in the Tamm-Dancoff approximation (TDA) using a Becke integration grid and a ZlmFit grid of normal quality. Scalar relativistic effects were included in the zeroth-order regular approximation (ZORA) and spin-orbit couplings (SOCs) were calculated perturbatively.

| Atom | x [Å] | y [Å] | z [Å] |
|---|---|---|---|
| C | 3.580 | 0.180 | −0.000 |
| C | 2.619 | 1.160 | −0.000 |
| C | 1.246 | 0.820 | 0.000 |
| C | 0.878 | −0.561 | 0.000 |
| C | 1.895 | −1.549 | 0.000 |
| C | 3.217 | −1.185 | 0.000 |
| C | 0.223 | 1.805 | −0.000 |
| C | −0.489 | −0.907 | 0.000 |
| C | −1.437 | 0.084 | −0.000 |
| C | −1.098 | 1.454 | −0.000 |
| H | 0.504 | 2.851 | −0.000 |
| H | 4.629 | 0.451 | 0.000 |
| H | 2.900 | 2.206 | −0.000 |
| H | 1.610 | −2.595 | 0.000 |
| H | 3.990 | −1.943 | 0.000 |
| H | −0.784 | −1.947 | 0.000 |
| H | −1.877 | 2.201 | −0.000 |
| N | −2.850 | −0.291 | −0.000 |
| O | −3.686 | 0.605 | −0.001 |
| O | −3.143 | −1.481 | 0.001 |

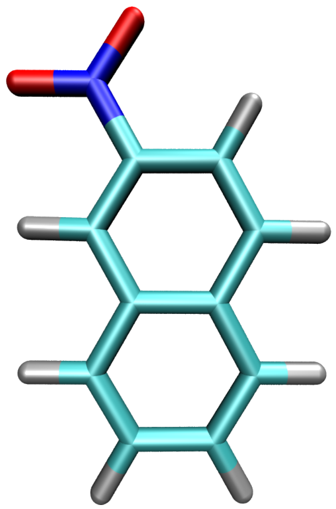

Figure S1: Ground-state minimum-energy geometry of 2NN in gas phase optimized at the PBE0/DZP level of theory.

**Absorption Spectrum.** Using the geometries of the initial conditions (see above), the absorption spectrum and the density of states (Figure S2) were simulated by Gaussian convoluting the oscillator-weighted and unweighted stick spectra, respectively, using a FWHM of 0.1 eV. Good agreement to experimental reference spectra can be obtained for 2NN in methanol (MeOH) using PBE0.[10] Up to our knowledge, there is no experimental absorption spectrum of 2NN in gas phase; in lieu, we show in Figure S2 the experimental absorption spectrum of 2NN recorded in n-heptane,[11] where the good agreement between the positions and intensity ratios of the calculated and experimental absorption bands over a large energy range is apparent. We note that the calculated spectrum does not show the fine structure of the experimental absorption bands. We encountered a similar situation in our study of 2NN in MeOH; there, however, we could explain the appearance of a shoulder in the third experimental absorption band by the presence of different electronic states, even though the shoulder was not visible in our calculated spectrum.[10] Thus, we believe the absence of experimentally visible features of the absorption bands in our calculated spectra is not a bad sign, and PBE0 does provide a good description of the electronic structure of 2NN.

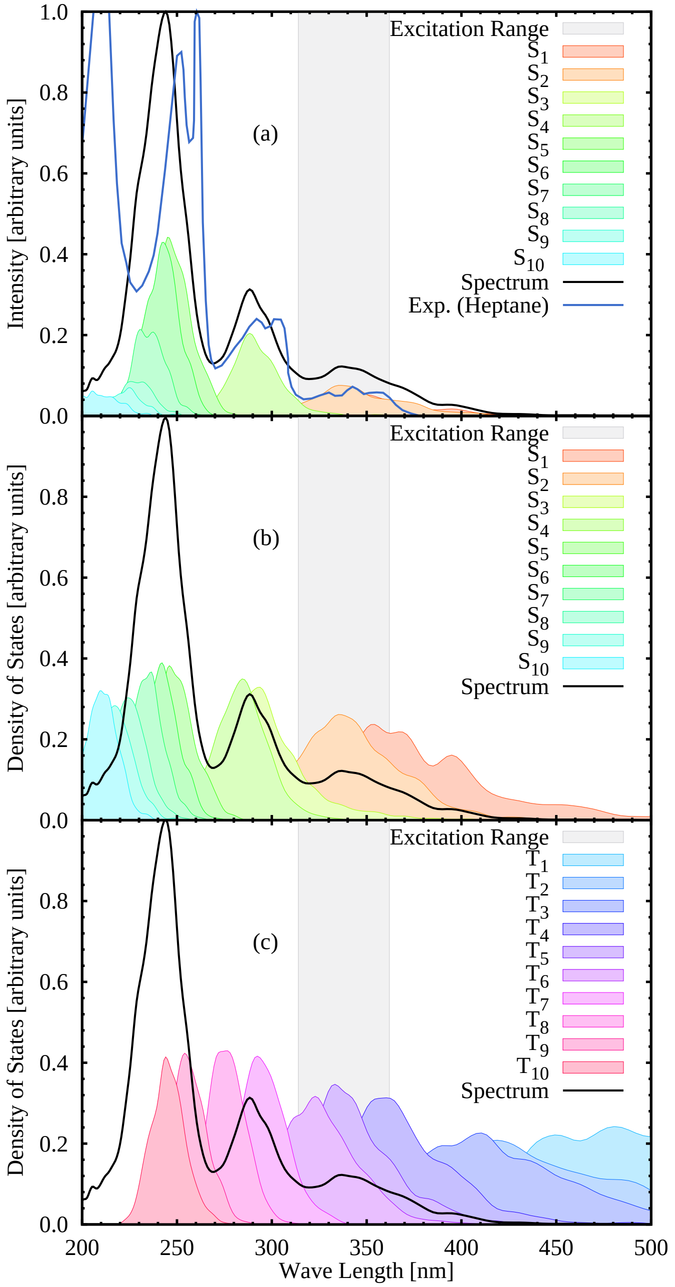


Figure S2: (a) Experimental absorption spectrum of 2NN in n-heptane(blue line),[11] calculated absortion spectrum in gas phase (black line), as well as individual state contributions to the absorption spectrum. (b/c) Calculated density of singlet/triplet states (filled curves) and absorption spectrum (black line). The grey area denotes the excitation window in the SHARC simulations. Calculations were performed using a thermal Wigner distribution at $T = 300$ K.

**Trajectories Set-Up.** 105 trajectories were set-up from the $T = 300$ K ensemble. For 2NN, ultrafast intersystem crossing has been observed in transient absorption experiments after excitation close to the maximum of the lowest-energy absorption band.[12] Similarly, we chose initial conditions to setup the trajectories stochastically as proposed in Ref. 13 in a 0.5 eV window around the maximum of the first absorption band (3.43-3.93 eV, see gray areas in Figure S2). Accordingly, 51 trajectories were started in the $S_1$ state and 54 trajectories were started in the $S_2$ state. Based on the density-of-states at the initial conditions, three singlet states ($S_0$, $S_1$, and $S_2$) and six triplet states ($T_1$-$T_6$) were included in the SHARC simulations and two additional singlet and triplet states were calculated as inactive states. To illustrate the characters of the excited-states included in the dynamics simulation, we show their corresponding states calculated at the FC geometry in Table S1. As mentioned in the main paper, one should keep in mind that the character of the states in the ensemble can differ from the FC excited states due to vibrational motion.

**SHARC Simulations.** Nonadiabatic dynamics simulations were performed for the 105 trajectories for a total simulation time of 500 fs. The nuclear time step was 0.5 fs and the electronic Schrödinger equation was integrated with a time step of 0.02 fs using the local diabatization method.[14] An energy-based decoherence correction with a constant of $C = 0.1$ a.u. was used[15,16] for the spin-adiabatic states. To save computation time, gradients for the non-adiabatic couplings were only calculated for electronic states with an energy gap less than 0.3 eV. Otherwise, the defaults of the SHARC program package and the recently implemented SHARC/ADF interface[8] have been used. From the subsequent analysis, six trajectories had to be excluded from the ensemble, as either the trajectory crashed prior to reaching the 500 fs simulation time or it showed intruder state problems that would have required the inclusion of more active electronic states in the simulations.

Table S1: Energies in eV, oscillator strengths $f_{OSC}$, electronic characters, and natural transition orbitals calculated at PBE0/DZP level of theory in gas phase describing the low-lying excited states of 2NN at the Franck-Condon geometry. Subscripts CT and LE denote charge-transfer and local excitation character, respectively.

| State | Energy (eV) | $f_{OSC}$ | Character | Natural Transition Orbitals |
|---|---|---|---|---|
| $S_1$ | 3.80 | 0.064 | $S_{CT}(\pi\pi^*)$ | 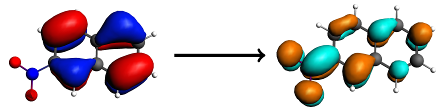 |
| $S_2$ | 3.85 | 0.000 | $S_{LE}(n\pi^*)$ | 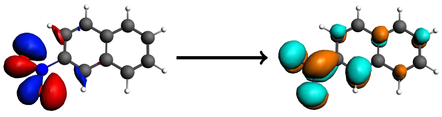 |
| $T_1$ | 2.85 | 0.000 | $T_{CT}(\pi\pi^*)$ | 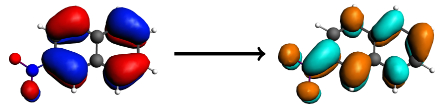 |
| $T_2$ | 3.32 | 0.000 | $T_{LE}(n\pi^*)$ | 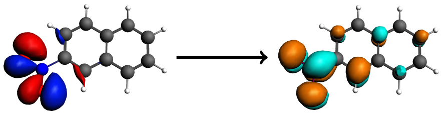 |
| $T_3$ | 3.32 | 0.000 | $T_{LE}(\pi\pi^*)$ | 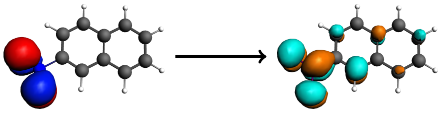 |
| $T_4$ | 3.67 | 0.000 | $T_{LE}(\pi\pi^*)$ | 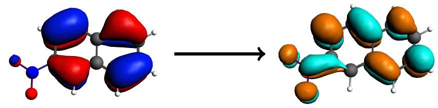 |
| $T_5$ | 3.77 | 0.000 | $T_{CT}(\pi\pi^*)$ | 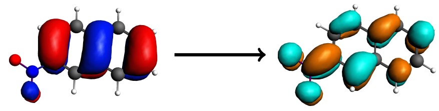 |
| $T_6$ | 4.00 | 0.000 | $T_{LE}(n\pi^*)$ | 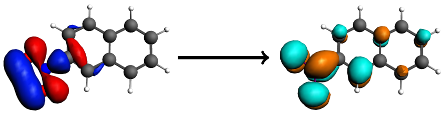 |

## S1.2 Problems Encountered in the Simulations

As mentioned above, a small number of the calculated trajectories had to be excluded for the analysis. Five trajectories from the $T = 300$ K ensemble were excluded as they crashed prematurely ($t < 332$ fs); all other trajectories could be propagated until $t = 500$ fs. An additional trajectory was excluded because it hopped to a (previously) inactive state*. The remaining 99 trajectories were considered for the analysis.

Additionally, we comment on two critical cases encountered for a small number of trajectories. The first is given by trajectories that exhibit a "unusually" large fluctuation of the total energy which occured in some cases where the $S_0$ and $S_1$ were nearly degenerate. Then the total energy could increase momentarily by up to 0.2-0.5 eV before it fell back to the previous range after the states separate in energy again. An example of this situation is shown in Figure S3(a), and one may speculate that it is due to the single-reference nature of TD-DFT that can only give an incomplete description of the multi-configurational nature that the ground state $S_0$ ingests when it is nearly degenerate with the $S_1$.† We note, however, that this is not the general behavior when the $S_0$ and the $S_1$ are in close in energy, as can be seen in the exemplary situation depicted in Figure S3(b) where the total energies remains almost constant in the near-degeneracy region. In total, we found the large energy fluctuation due to a $S_1/S_0$ near-degeneracy for 8 trajectories. We did not exclude these trajectories from the analysis for the following reasons. First, the DFT/TDDFT calculations do converge in this region of the potential energy surface (PES), and as the total energy goes back to the range outside of this region, we can conclude that the the trajectory can safely leave this region. And second, one may speculate that a trajectory entering the region of near-degeneracy should hop to the ground state; however, absence of this event does not indicate an error as it is in general possible for a trajectory to hop to

*the SHARC program is able to follow the character of the MCH states by monitoring wave function overlaps[17] of the calculated states

†the trajectory in Figure S3(a) actually represents the case with the largest fluctuation in total energy of all trajectories (0.46 eV)

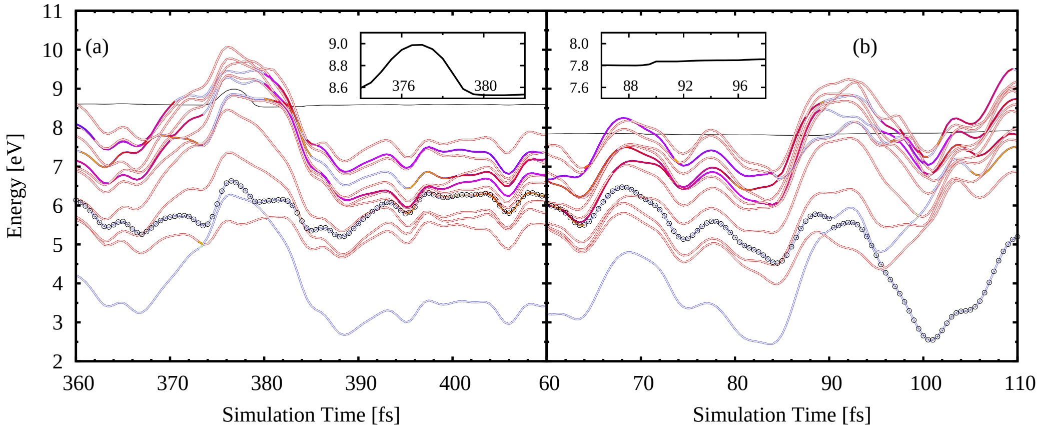


Figure S3: Total energy (black line) and energies of the MCH states (colored lines) in eV of two trajectories showing a $S_1/S_0$ near-degeneracy. The circles denote the active state in the simulation. Insets show a magnification of the total energies in the near-degeneracy regions.

the ground state without showing the fluctuation in the total energy, see Figure S3(b). Potential problems that TDDFT surface hopping can exhibit in the region of $S_1/S_0$ near degeneracies are discussed by Tavernelli and co-workers in Ref. 18.

The second critical case is provided by situations where hops occur between states with a large energy gap ($>$ 1 eV). An example is shown in Figure S4. There, a trajectory is in the $S_1$ state until $t = 204.5$ fs, when it hops to the $S_2$ state which lies 1.63 eV higher in energy. In order to conserve the total energy of the trajectory, this sudden jump in potential energy is compensated by a rescaling of the momenta of the system decreasing the kinetic energy, as can also be seen in Figure S4. The rescaling is performed by changing the momenta of all atoms in the same manner, i.e., via multiplication by the same factor so that the total energy is conserved. The probability for the large energy hop is very small amounting only to $P \sim 0.001$, however, this hop still occured.[‡] For such an event to be statistically significant, one should propagate at least $N = 1/P$ trajectories –so that the event occurs stochastically once. Such low-

[‡]to decide whether or not a hop occurs, a random number $\in [0, 1]$ is sampled and compared to the hopping probability; when the probability is larger than the random number, the trajectory hops to a different state

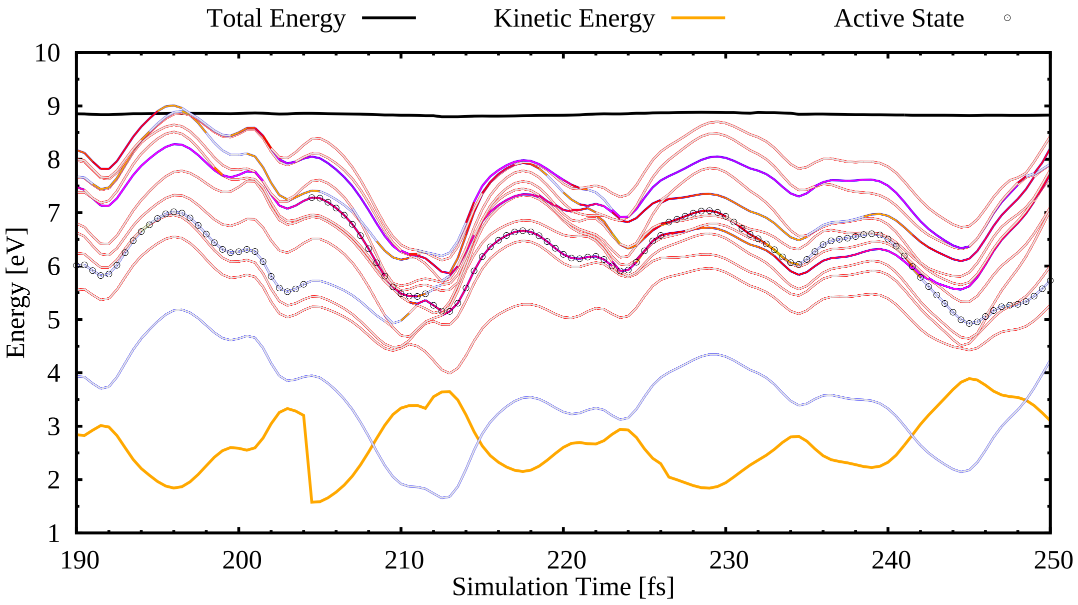


Figure S4: Total energy (black line), kinetic energy (orange line), and energies of the MCH states (colored lines) in eV of a trajectories showing a hop between states with a large energy gap. The circles denote the active state in the simulation.

probability hops occurred 12 times. Despite this is a considerably large number for the comparatively small numbers of trajectories ($N = 99$), we believe that the occurrence of the low-probability hops does not have a significant impact on our results. All low-probabilities hops occurred either from the $S_1$ or the $T_1$ to higher-lying excited singlet and triplet states, respectively. Before the low-probability hop, most of the trajectories had been for a considerable simulation time in the $S_1$ or $T_1$ state, and this (initial) state was quickly retrieved within the remaining simulation time (average retrieval time $\tau_{\text{ret}} = 18$ fs). Thus, we can safely include these trajectories with low-probability hops in our analysis.

## S2 Mechanism of Excited-State Dynamics

### S2.1 Major Reaction Channels

Figure S5 shows the time evolution of the populations of the MCH states of 2NN in gas phase. Figure 2 in the main paper is similar, but shows only the sum of the excited triplet state populations $T_n$ ($n$ = 2-6) and the lowest-energy triplet state $T_1$, allowing for a general reaction mechanism, where the population is transferred from the $S_1$ to some higher-lying triplet state $T_n$ before subsequent deactivation to $T_1$. This can be

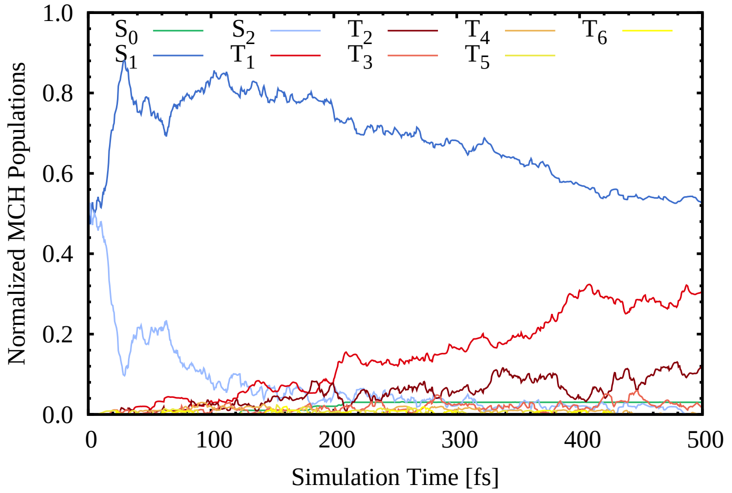


Figure S5: Time evolution of the state populations in the first 500 fs of the excited-state dynamics of 2NN.

seen explicitely when analyzing the hops between the different MCH states that occur during the dynamics simulation. Thus, we show the total amount of hops of all 99 trajectories during the 500 fs simulation time in Table S2(a), e.g., showing 280 hops from $S_2$ to $S_1$ and 234 hops from $S_1$ to $S_2$, from which we calculated also the net amount of hops between the MCH states (Table S2(b)). The net amount of hops directly show from which MCH state population is transferred to another MCH state, e.g., it shows that population from the $S_2$ is almost completetly transferred to the $S_1$ state (46 net hops), while only a minor part is transferred to the triplet states ($2 + 2$ hops).

Table S2: Total amount (a) and net amount (b) of hops between the different MCH states in the SHARC simulations after 500 fs. The most important net hops have been highlighted by color.

| (a) | $S_0$ | $S_1$ | $S_2$ | $T_1$ | $T_2$ | $T_3$ | $T_4$ | $T_5$ | $T_6$ |
|---|---|---|---|---|---|---|---|---|---|
| $S_0$ | 1993 | 4 | 0 | 3 | 0 | 0 | 0 | 0 | 0 |
| $S_1$ | 1 | 66270 | 280 | 0 | 234 | 108 | 30 | 6 | 0 |
| $S_2$ | 0 | 234 | 6723 | 0 | 0 | 0 | 16 | 18 | 8 |
| $T_1$ | 3 | 0 | 0 | 13845 | 118 | 16 | 1 | 0 | 0 |
| $T_2$ | 0 | 259 | 0 | 87 | 5316 | 70 | 7 | 0 | 0 |
| $T_3$ | 0 | 122 | 0 | 17 | 50 | 1614 | 13 | 2 | 0 |
| $T_4$ | 0 | 32 | 18 | 2 | 3 | 7 | 743 | 16 | 3 |
| $T_5$ | 0 | 6 | 20 | 0 | 2 | 1 | 11 | 327 | 16 |
| $T_6$ | 0 | 0 | 8 | 0 | 2 | 0 | 3 | 14 | 259 |

| $S_2 \xrightarrow{k_S} S_1$ | $S_1 \xrightarrow{k_{ISC}} T_n$ | $T_n \xrightarrow{k_T} T_1$ |
|---|---|---|

| (b) | $S_0$ | $S_1$ | $S_2$ | $T_1$ | $T_2$ | $T_3$ | $T_4$ | $T_5$ | $T_6$ |
|---|---|---|---|---|---|---|---|---|---|
| $S_0$ | – | 3 | 0 | 0 | 0 | 0 | 0 | 0 | 0 |
| $S_1$ | −3 | – | 46 | 0 | −25 | −14 | −2 | 0 | 0 |
| $S_2$ | 0 | −46 | – | 0 | 0 | 0 | −2 | −2 | 0 |
| $T_1$ | 0 | 0 | 0 | – | 31 | −2 | −1 | 0 | 0 |
| $T_2$ | 0 | 25 | 0 | −31 | – | 20 | 4 | −2 | −2 |
| $T_3$ | 0 | 14 | 0 | 2 | −20 | – | 6 | 1 | 0 |
| $T_4$ | 0 | 2 | 2 | 1 | −4 | −6 | – | 5 | 0 |
| $T_5$ | 0 | 0 | 2 | 0 | 2 | −1 | −5 | – | 2 |
| $T_6$ | 0 | 0 | 0 | 0 | 2 | 0 | 0 | −2 | – |

Table S3: Rate constants $k_i$ (in fs$^{-1}$) as well as corresponding mean time constants $\tau_i = 1/k_i$ and errors $\Delta\tau_i$ (in fs) calculated using the bootstrap method for the time evolution of the MCH populations according to the mechanism shown in eq. (1).

| Reaction | $\mathbf{k}^a$ | $\boldsymbol{\tau}^a$ | $\langle\boldsymbol{\tau}\rangle^b$ | $\boldsymbol{\Delta\tau}^b$ | $\langle\boldsymbol{\tau}\rangle^c$ | $\boldsymbol{\Delta\tau}^c$ |
|---|---|---|---|---|---|---|
| $\mathbf{S_2} \xrightarrow{\mathbf{k_S}} \mathbf{S_1}$ | 0.0181936 | 55.0 | 56.3 | ±8.3 | 55.7 | +9.0/ − 7.7 |
| $\mathbf{S_1} \xrightarrow{\mathbf{k_{ISC}}} \mathbf{T_n}$ | 0.0013640 | 733.1 | 710.7 | ±100.8 | 703.8 | +105.4/ − 91.6 |
| $\mathbf{T_2} \xrightarrow{\mathbf{k_T}} \mathbf{T_1}$ | 0.0071099 | 140.6 | 148.9 | ±19.4 | 147.7 | +20.4/ − 17.9 |

[a] obtained from fit of the populations shown in Figure 2 in the main paper
[b] arithmetic mean and error of 100 bootstrap copies
[c] geometric mean and error of 100 bootstrap copies

Based on the excited-state populations and the hops between the different MCH states, we proposed the (simplified) kinetic model

$$S_2 \xrightarrow{k_S} S_1 \xrightarrow{k_{ISC}} T_n \xrightarrow{k_T} T_1 \quad (1)$$

Following eq. (1), we have calculated functions for the time evolution of the $S_2/S_1/T_n/T_1$ populations using a nonlinear least-squares fit, which are shown in Figure 2 in the main paper. The rate constants $k_S/k_{ISC}/k_T$ and corresponding time constants $\tau_i = 1/k_i$ obtained in these fits are shown in Table S3. For each time constant we have also calculated error margins using the bootstrap method as suggested by Truhlar et al.[19]

The time constants obtained by the single fit of the populations as well as the arithmetic and geometric means in the bootstrap calculation are very similar for each reaction. Thus, we report only the arithmetic mean and its error margin in the discussion in the main paper. Here, we only mention that the error for each time constant amounts to ca. 15 % of the corresponding time constant's value. Thus, we have sampled a sufficient number of trajectories to unambiguously describe our proposed reaction mechanism [see eq. (1)] as the time constants of consecutive reactions are always of a different order of magnitude, i.e., $\tau_S \ll \tau_{ISC}$ and $\tau_{ISC} \gg \tau_T$.

## S2.2 Minor Reaction Channels

The mechanistic model of eq. (1) only included the major reaction channels in the first 500 fs of the excited-state dynamics of 2NN. The model does not include direct relaxation from the $S_2$ to the triplet states, the relaxation to the ground state $S_0$, and the individual relaxation processes within the manifold of the higher-lying triplet states $T_n$ ($n$ = 2-6). The direct $S_2 \rightarrow T_n$ relaxation is a minor channel populating the triplet states compared to the major route $S_2 \rightarrow S_1 \rightarrow T_n$ (see hops in Table S2). Based on our simulations, for this minor channel the time constant§ would amount to $\tau = 1099 \pm 8540$ fs, i.e., possessing a huge error due to the small number of trajectories. Since this time constant is not reliable, we can only conclude that the $S_2 \rightarrow T_n$ process is much slower that the competing $S_2 \rightarrow S_1$ process. Likewise, the relaxation to the ground state $S_0$ from the $S_1$ state is also not well represented by a sufficient number of trajectories and a time constant is meaningless, as it occurs on a slower time scale than our simulation time ($\tau_{S_1 \rightarrow S_0} = 10205 \pm 10629$ fs.§ ). Also note that due to the small number of trajectories between the individual $T_n$ states, it is not possible to calculate individual reaction rates without problems of overfitting. Instead, we resort to describe intersystem crossing from (mainly) the $S_1$ state to the higher-lying triplet states $T_n$ ($n = 2$-6).

## S2.3 ISC Pathways

In the main paper, we stated that ISC occurs via two different pathways, a $^1n\pi^* \rightarrow^3 \pi\pi^*$ route and a $^1\pi\pi^* \rightarrow^3 n\pi^*$ route. This was established after manual inspection of the natural transition orbitals of the singlet and triplet excited states mediating the ISC process for a small fraction of all ISC hops occurring in the dynamics.¶ To subsequently analyze all ISC hopping events, we calculated the mean atomic electron/hole

§arithmetic mean of 100 bootstrap copies with error margins

¶the small fraction consisted of ca. 30 ISC hops out of a total of 418 ISC hops occurring in all trajectories

difference population of the oxygen atoms of the nitro group for all singlet states using the TheoDORE program package.[20–23] If the difference was negative, i.e., electron density at the oxygen is lower in the excited state than in the ground state, the state was characterized as the $^1n\pi^*$ state, whereas if the difference was positive, it was characterized as the $^1\pi\pi^*$ state (see Figure 3(b) in the main paper). The character of the receiver triplet state for the ISC follows the El-Sayed's rules,[24] which we verified for the fraction of ISC hops that we inspected individually.

Having characterized the ISC hops as either $^1n\pi^* \rightarrow^3 \pi\pi^*$ or $^1\pi\pi^* \rightarrow^3 n\pi^*$, we have calulated the mean atomic hole/electron difference populations for both sets of states which are shown in Figure 4 in the main paper. Alongside we also show the main natural transition orbitals (NTOs) involved in the excited states at the FC geometry, which resembled the main NTOs describing the excited states that we inspected individually. Note that the actual NTOs in the transition differ from those depicted in Figure 4 to a certain degree, e.g., because of the distortion of the structures due to admixture with other orbitals. As such, the NTOs in Figure 4 in the main paper are shown only for illustration purposes. As the structures are taken from the dynamics simulations, naturally they differ from each other and from the optimized FC geometry. Thus it is not straightforward to define, e.g., average NTOs that describe different (although similar) excitations at different points of the trajectories. In contrast, the hole/electron difference populations are atomic properties that can easily be averaged over different structures and are therefore more convenient to analyze. Note that, unfortunately, as atomic properties, the hole/electron populations do not contain any "directional" information, i.e., they do not allow a direct discrimination between $n\pi^*$ and $\pi\pi^*$ states if the $n$ and $\pi$ orbitals are located at the same atoms –see, e.g., the similar populations for the $T_{LE}(\pi'\pi^*)$ and $T_{LE}(n\pi^*)$ states in Figure 4 in the main paper. However, having verified El-Sayed's rules at a sizeable number of excited states in the ISC hops, we feel assured to apply the rule generally, allowing us to safely differentiate all $^1n\pi^* \rightarrow^3 \pi\pi^*$ from $^1\pi\pi^* \rightarrow^3 n\pi^*$ hops.

Based on the discrimination of the two ISC pathways, we determined their importance in the excited-state dynamics. Their relative contribution was shown in Figure 4 in the main paper, which was calculated as the fraction of trajectories that entered the triplet manifold via the respective pathway and stayed in the a triplet state after a simulation time of 500 fs. These numbers are collected in Table S4. The actual number of trajectories ending up in a triplet state via the minor pathway is small, i.e., 4 trajectories, raising the question on the statistical significance (see above). Since the ratio for the contributions of the minor and major pathway is similar within the number of trajectories (ca. 9 %) and the number of hops (ca. 12 %), we believe that the relative contributions of the minor and major pathways determined by the number of trajectories ending in a triplet represent an acceptable estimate.

The total numbers of hops from singlet to triplet states are much larger than the number of trajectories ending up in a triplet state as it occurs quite frequently that a trajectory hops from a singlet to triplet state in one time step only to hop back to a singlet state at the next time step. Thus, a trajectory needs in average ca. 10 "attempts" to hop from a singlet to a triplet state in order to *remain* in a triplet state.

Table S4: Number of trajectories in triplet states and number of hops to triplet states as well as contributions of the major and minor ISC pathways after a simulation time of 500 fs.

| **Number of Trajectories** | | |
|---|---|---|
| $N_{\text{traj}}$ in triplet states | 46 | |
| ... via major pathway | 42 | (91.3 %) |
| ... via minor pathway | 4 | (8.7 %) |
| Total number of trajectories | 99 | |
| $N_{\text{hops}}$ to triplet states | 418 | |
| ... via major pathway | 369 | (88.2 %) |
| ... via minor pathway | 49 | (11.8 %) |

## S2.4 Spin-Orbit Couplings in the ISC Pathways

In this section we include a brief discussion of the SOCs during the dynamics. For this, we first show the time evolution of two trajectories undergoing ISC via the major and minor pathway (Figure S6) along with the size of the SOCs between the $S_1$ and the lowest-lying triplet states $T_n$. The SOCs were calculated as the vector sum of the SOC matrix element between the $S_1$ singlet state and all three components of the respective triplet state, i.e.,

$$\mathrm{SOC}(S_1, T_n) = \sqrt{\left|\langle S_1|\mathcal{H}_{SOC}|T_n^{M_S=1}\rangle\right|^2 + \left|\langle S_1|\mathcal{H}_{SOC}|T_n^{M_S=0}\rangle\right|^2 + \left|\langle S_1|\mathcal{H}_{SOC}|T_n^{M_S=-1}\rangle\right|^2} \tag{2}$$

The trajectories in Figure S6 were selected as they show a $S_1$-$T_n$ hop very early in the simulation time and afterwards stay for the remaining simulation in the triplet manifold; we only show an interval of 20 fs around the hopping event.

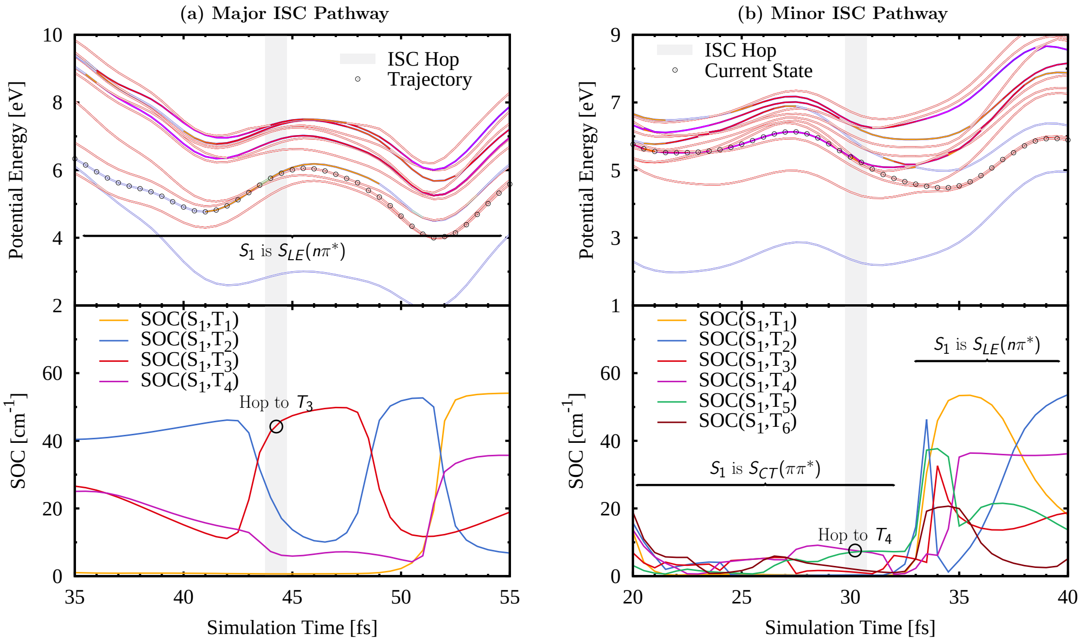


Figure S6: Time evolution of spin-orbit couplings between the $S_1$ and the lowest-lying triplet states $T_n$ for a trajectory undergoing ISC via the (a) major pathway or (b) minor pathway. The grey areas denote the time steps between which ISC hops occur.

Before the ISC hop, the trajectory undergoing ISC via the major pathway [Figure S6(a)] is in the $S_1$ state which can be characterized as the $S_{LE}(n\pi^*)$ state. For this trajectory, two ($T_1$, $T_2$) or three ($T_1$-$T_3$) triplet states are close in energy. For one of the triplet states, the SOC with this singlet state is considerably large ranging from 40-50 cm$^{-1}$. In the MCH electronic state representation, this triplet state is the $T_2$ until ca. 43 fs before it changes place in the energetic ordering and becomes the $T_3$ state; later this state briefly becomes the $T_2$ again and finally becomes the $T_1$ at ca. 53 fs. In terms of character, this triplet state is the locally excited $T_{LE}(\pi\pi^*)$ state to which the trajectory hops at 44.4 fs. At this point, the SOC is of the same size as the average value of SOCs reported for the major pathway in the main paper, i.e., SOC $\sim$ 40 cm$^{-1}$ (see Figure 4).

The trajectory undergoing ISC via the minor pathway [Figure S6(b)] initially is in the $S_1$ which is the $S_{CT}(\pi\pi^*)$ state. Although there are several triplet states close in energy to this singlet state, the SOCs are considerably smaller ($<$ 10 cm$^{-1}$) than for the trajectory undergoing ISC via the major pathway. Still, eventually at 30.5 fs, the trajectory hops from the $S_1$ state to the $T_4$ state, which is the $T_{LE}(n\pi^*)$ state. The SOC during this hop is 9 cm$^{-1}$ which is again of similar size as the average value of SOCs reported for the minor pathway in the main paper, i.e., SOC $\sim$ 8 cm$^{-1}$ (see Figure 4). Shortly, after the trajectory has hopped into the triplet manifold, the MCH singlet states change in character and the $S_{LE}(n\pi^*)$ becomes the $S_1$ at 33 fs. This state possesses larger SOCs with the low-lying triplet states and explains the sudden increase in $S_1$-$T_n$-SOCs shown in Figure S7.

The size of the SOCs is related to the probability of singlet-to-triplet ISC occuring via the minor or major pathway, but it is also related to the probability for the trajectory to hop back from the triplet to the singlet state. This usually occurs within a few time steps after the singlet-triplet hop, where the trajectory is still in the same region of the potential energy surface, meaning that the energy gaps and SOC between the states are approximately the same and, thus, so is the hopping probability. To

demonstrate this, we show in Figure S7 the time evolution of a trajectory that hops back and forth six times between singlet and triplet states during the last 200 fs of the 500 fs simulation time.

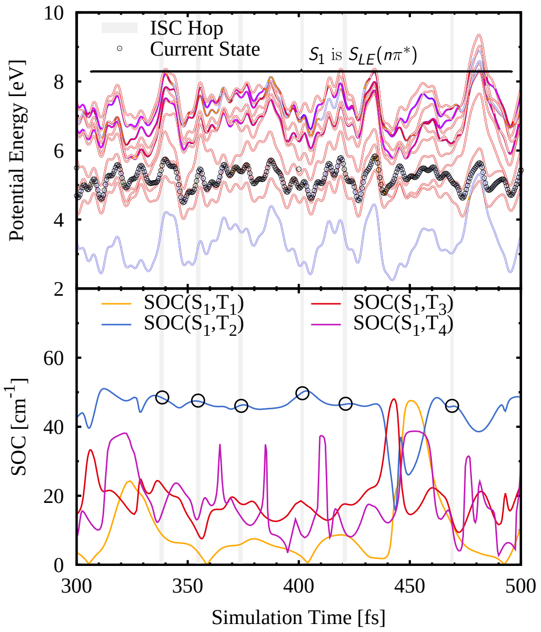


Figure S7: Time evolution of spin-orbit couplings between the $S_1$ and $T_n$ ($n = 1$-4) for a trajectory hopping back and forth between the $S_1$ and $T_2$ in the major pathway. The gray areas denote the time steps between which ISC hops occur.

The trajectory initially is in the $S_{LE}(n\pi^*)$ state and possesses a large SOCs of ca. 50 cm$^{-1}$ with the $T_2$ or $T_{LE}(\pi\pi^*)$ state. Although the trajectory hops several times, it returns within 1 or 2 time steps back to the singlet state where it finishes at the end our simulation. This example demonstrates that a large SOC is not sufficient for ISC, but the trajectory must also be able to quickly escape the singlet-triplet hopping region while being in the triplet state.

## S2.5 Initial Dynamics in the Singlet States

In the main paper, we analyze the $S_{CT}(\pi\pi^*) \rightarrow S_{LE}(n\pi^*)$ dynamics. To do so, we discriminate the states as either $S_{CT}$ or $S_{LE}$ by their dipole moment $\mu$ using $\mu > 8$ D

for $S_{CT}$ and $\mu < 8$ D for $S_{LE}$, respectively. The arbitrary value of 8 D is chosen because it lies in between the initial range of dipole moments (ca. 10-12 D) and the value towards which the ensembles converges ($\mu = 4$ D, see Figure 5(a) in the main paper). Using $\mu = 8$ D as a criterion to distinguish the states $S_{CT}$ and $S_{LE}$, we obtained the time constant of $\tau = 81$ fs for the population transfer of $S_{CT} \rightarrow S_{LE}$. Despite the arbitrariness of $\mu = 8$ D, we note that slightly different reasonable choices of $\mu$ give similar values for $\tau$, e.g., ranging from 90 fs ($\mu = 6$ D) to 78 fs ($\mu = 9$ D). We consider these values reasonable as they also lie between the dipole moments the $S_{CT}(\pi\pi^*)$ and $S_{LE}(n\pi^*)$ states at their optimized geometries (Table S5).

Table S5: Coordinates in Å of the optimized geometries of the $S_{CT}(\pi\pi^*)$ and $S_{LE}(n\pi^*)$ states as well as their energies and dipole moments.

| | | $S_{CT}(\pi\pi^*)$ | | | $S_{LE}(n\pi^*)$ | |
|---|---|---|---|---|---|---|
| | **X** | **Y** | **Z** | **X** | **Y** | **Z** |
| C | 3.608 | 0.185 | 0.000 | 3.582 | 0.192 | 0.011 |
| C | 2.624 | 1.156 | −0.000 | 2.611 | 1.159 | 0.006 |
| C | 1.248 | 0.809 | −0.000 | 1.252 | 0.812 | 0.002 |
| C | 0.863 | −0.562 | −0.000 | 0.885 | −0.564 | 0.003 |
| C | 1.878 | −1.519 | 0.000 | 1.909 | −1.539 | 0.008 |
| C | 3.230 | −1.151 | 0.000 | 3.224 | −1.166 | 0.013 |
| C | 0.243 | 1.777 | −0.000 | 0.227 | 1.795 | −0.002 |
| C | −0.503 | −0.919 | −0.000 | −0.467 | −0.921 | 0.000 |
| C | −1.480 | 0.058 | 0.000 | −1.434 | 0.075 | −0.004 |
| C | −1.103 | 1.404 | −0.000 | −1.083 | 1.452 | −0.005 |
| H | 0.505 | 2.828 | −0.000 | 0.507 | 2.841 | −0.003 |
| H | 4.654 | 0.460 | 0.000 | 4.627 | 0.471 | 0.014 |
| H | 2.896 | 2.205 | −0.000 | 2.884 | 2.208 | 0.004 |
| H | 1.613 | −2.570 | 0.000 | 1.637 | −2.587 | 0.009 |
| H | 3.987 | −1.926 | 0.000 | 3.999 | −1.923 | 0.016 |
| H | −0.818 | −1.954 | 0.000 | −0.757 | −1.962 | 0.001 |
| H | −1.896 | 2.141 | −0.001 | −1.863 | 2.201 | −0.009 |
| N | −2.849 | −0.276 | 0.001 | −2.739 | −0.256 | −0.007 |
| O | −3.688 | 0.670 | −0.001 | −3.746 | 0.535 | −0.014 |
| O | −3.121 | −1.509 | 0.000 | −3.237 | −1.439 | −0.005 |
| E | | 3.30 eV | | | 1.93 eV | |
| $\mu$ | | 12.9 D | | | 3.7 D | |

In the above analysis, we assume that the initial dynamics takes place within the singlet states and thus all the population is in the singlet states. Figure S8 shows the average dipole moment for the singlet, triplet and all the states juxtaposed. As it can be seen, the average dipole moment of all trajectories $\langle \mu_{\text{all}}(t) \rangle$ (green line) resembles very closely to that of the singlet states $\langle \mu_S(t) \rangle$ (blue line), even at longer times when the triplet states already have gained substantial amount of population.

Figure S8: Time evolution of the average dipole moment $\langle \mu \rangle$ for all states/only singlet states/only triplet states as well as the singlet and triplet quantum population of all trajectories, respectively.

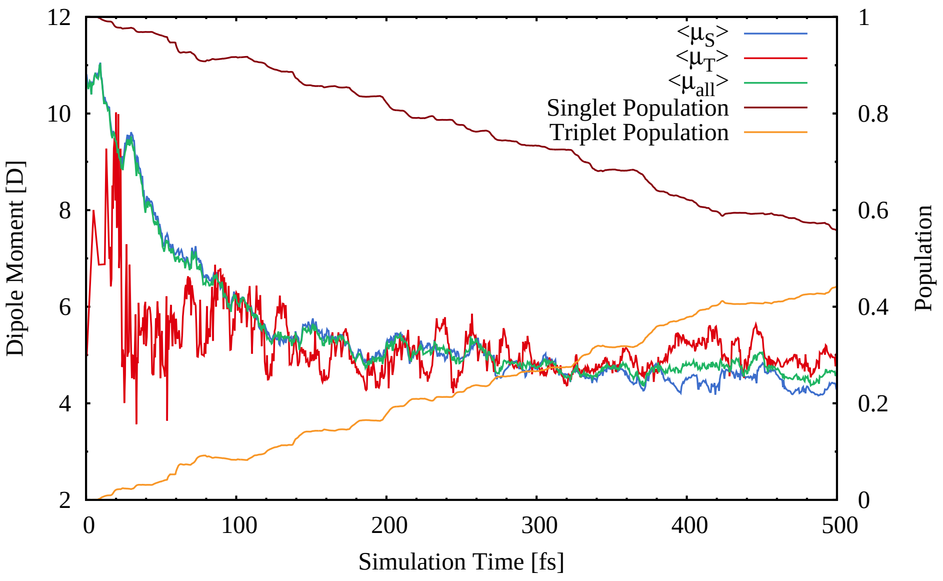


## S2.6 Normal Mode Analysis

Figure S9 shows the normal mode analysis (NMA)[25,26] for different sets of geometries: all geometries in the initial conditions (labelled as "Init"), the hopping geometries corresponding to the major/minor ISC pathway (labelled as "Major"/"Minor"), and the geometries where the active state is a singlet state with a dipole moment $\mu <$ 8 D/$\mu >$ 8 D (labelled as "$S_{LE}(n\pi^*)$"/"$S_{CT}(\pi\pi^*)$"). The corresponding vibrational frequencies are listed in Table S6.

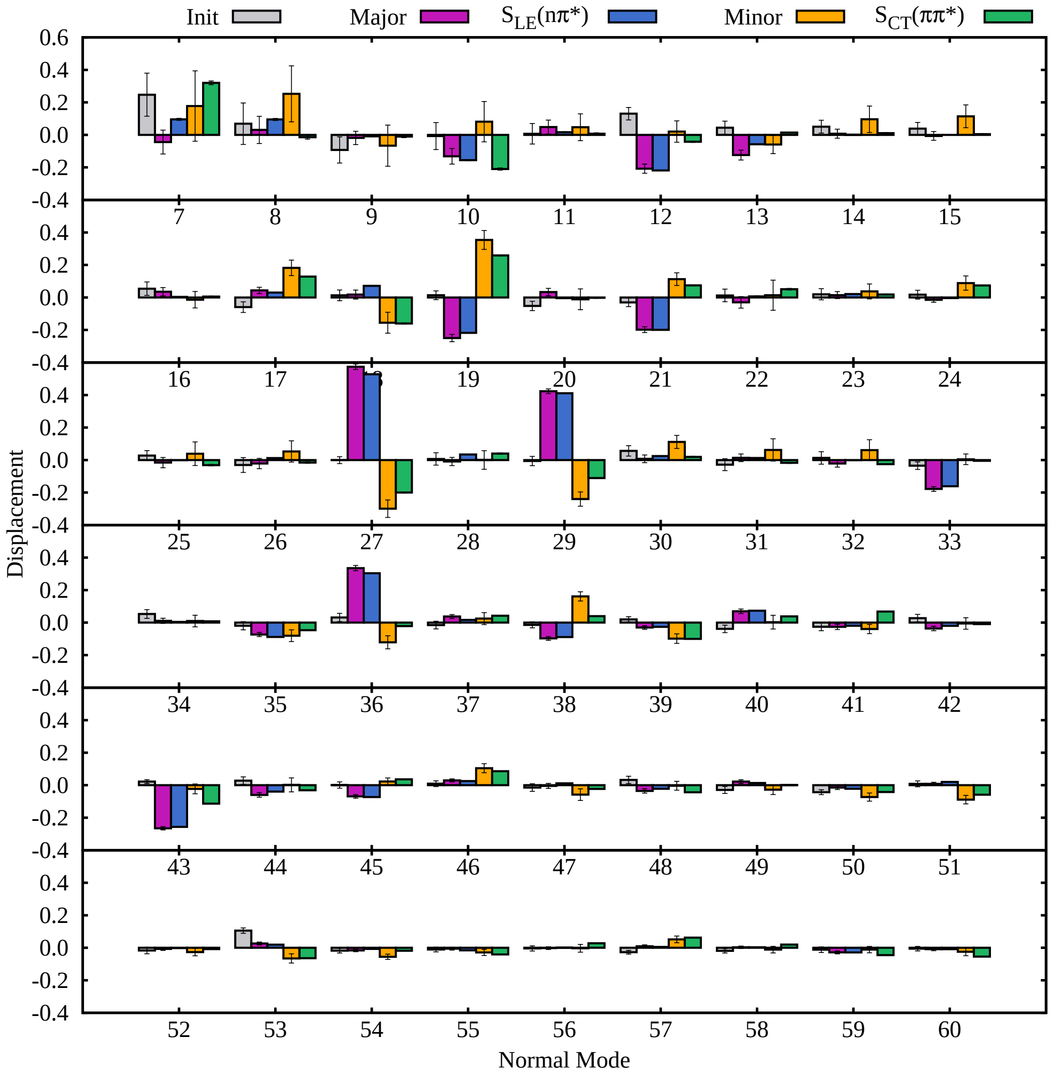


Figure S9: Normal mode analysis of the initial conditions, all hopping geometries in the major and minor pathway, and all geometries in the $^1n\pi^*$ and $^1\pi\pi^*$ states. Normal modes 1-6 refer to translation and rotation and are not depicted here. See Table S6 for the vibrational frequencies of all normal modes.

Table S6: Vibrational frequencies $\omega$ of normal modes in $cm^{-1}$.

| **Mode** | $\omega$ | **Mode** | $\omega$ | **Mode** | $\omega$ | **Mode** | $\omega$ | **Mode** | $\omega$ | **Mode** | $\omega$ |
|---|---|---|---|---|---|---|---|---|---|---|---|
| 7 | 87 | 16 | 500 | 25 | 794 | 34 | 982 | 43 | 1369 | 52 | 1641 |
| 8 | 103 | 17 | 526 | 26 | 823 | 35 | 1038 | 44 | 1378 | 53 | 1668 |
| 9 | 189 | 18 | 544 | 27 | 825 | 36 | 1096 | 45 | 1389 | 54 | 3198 |
| 10 | 199 | 19 | 611 | 28 | 878 | 37 | 1156 | 46 | 1406 | 55 | 3203 |
| 11 | 261 | 20 | 626 | 29 | 912 | 38 | 1166 | 47 | 1471 | 56 | 3209 |
| 12 | 339 | 21 | 650 | 30 | 918 | 39 | 1176 | 48 | 1485 | 57 | 3214 |
| 13 | 379 | 22 | 735 | 31 | 959 | 40 | 1225 | 49 | 1538 | 58 | 3224 |
| 14 | 399 | 23 | 773 | 32 | 972 | 41 | 1258 | 50 | 1584 | 59 | 3245 |
| 15 | 479 | 24 | 778 | 33 | 973 | 42 | 1288 | 51 | 1623 | 60 | 3261 |

Comparing the normal mode displacement (NMD) of the initial conditions (gray bars) with those of the ISC geometries of the major pathway (dark blue) and minor pathway (dark red) allows us to identify the decisive motion leading to ISC. With the exception of normal mode 7 –that describes mainly the nitro group torsion –all NMDs are very small for the initial geometries. As it can be seen, the normal modes with large displacement at the ISC geometries are the 19, 27, 29, 36, and 43 –summarized in Figure 6 of the main paper. For these normal modes, we show the displacement vectors in Figure S10.

## S2.7 Nuclear Motion

In the main paper we discuss the nuclear motion in terms of three internal coordinates that are responsible to drive the system towards intersystem crossing (recall Figure 6). Trajectories in the singlet or triplet state are discriminated with the spin expectation value of $\langle S^2 \rangle$; those in either the $S_{LE}(n\pi^*)$ or $S_{CT}(\pi\pi^*)$ state are discriminated with the dipole moment criterion as explained above.

Figure S11 shows the averages and standard deviations of the internal coordinates calculated for the hopping geometries and the tim evolultion of these values of the trajectories in the singlet and the triplet states. The standard deviations become quite large in the case where only a few trajectories are available at a given simulation

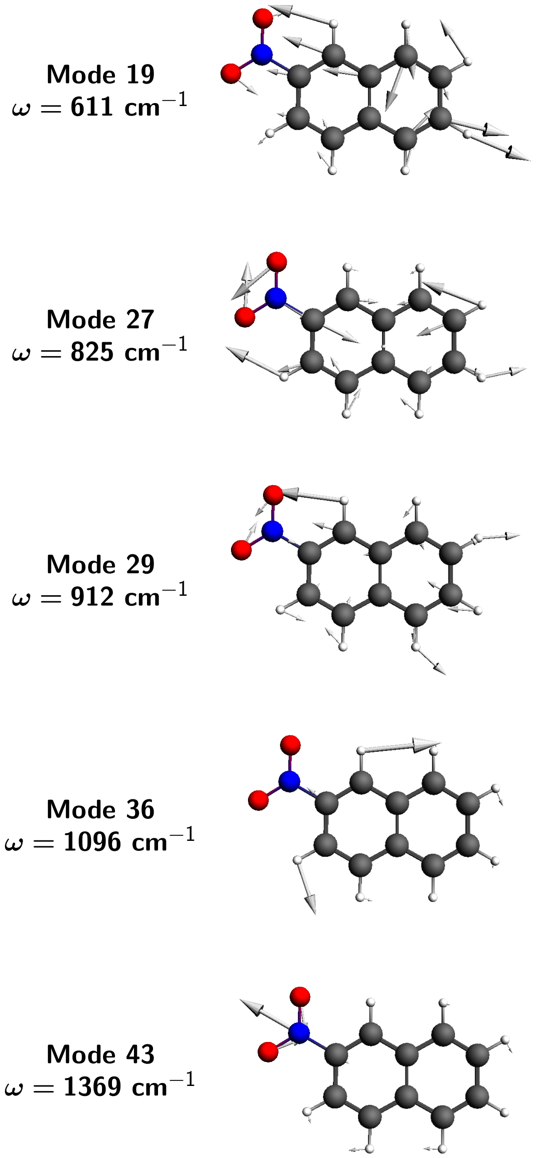


Figure S10: Displacement vectors of the important normal modes in the excited-state dynamics of 2NN.

time; this is e.g., the case at early times for the triplet states and at later times for the $S_{CT}(\pi\pi^*)$ state. Accordingly, caution is required to interpret the behavior of the average internal coordinates at these points. For completeness, the evolution of the torsional angle $\tau_{\mathrm{CCNO}}$ and its standard deviations are also shown in Figure S11, as the torsion of the nitro group has been discussed in Refs. 12, 27, 28. As it can be seen, the average of $\tau_{\mathrm{CCNO}}$ is never zero, but it is small, in agreement with the fact that no dissociation is observed for 2NN.

Note that the curves shown in both Figures 6(b-d) in the main paper and S11 are not pure averages but Bezier fits calculated with GNUPLOT. We have excluded 10 data points in the beginning of the simulation from the Bezier fit of the average internal coordinates of the triplet trajectories as there were no trajectories in a triplet state yet. In both Figures 6 and S11 we use the average of both bond distances $r_{\mathrm{NO}}$ and the four torsional angles $\tau_{\mathrm{CCNO}}$ that can be derived from the molecular structure.

# References


(1) Mai, S.; Richter, M.; Ruckenbauer, M.; Oppel, M.; Marquetand, P.; González, L. *SHARC: Surface Hopping Including Arbitrary Couplings – Program Package for Non-Adiabatic Dynamics*, sharc-md.org, 2014.

(2) Richter, M.; Marquetand, P.; González-Vázquez, J.; Sola, I.; González, L. *J. Chem. Theory Comput.* **2011**, *7*, 1253.

(3) Mai, S.; Marquetand, P.; González, L. *Int. J. Quant. Chem.* **2015**, *115*, 1215–1231.

(4) Adamo, C.; Barone, V. *J. Chem. Phys.* **1999**, *110*, 6158–6170.

(5) Ernzerhof, M.; Scuseria, G. E. *J. Chem. Phys.* **1999**, *110*, 5029–5036.

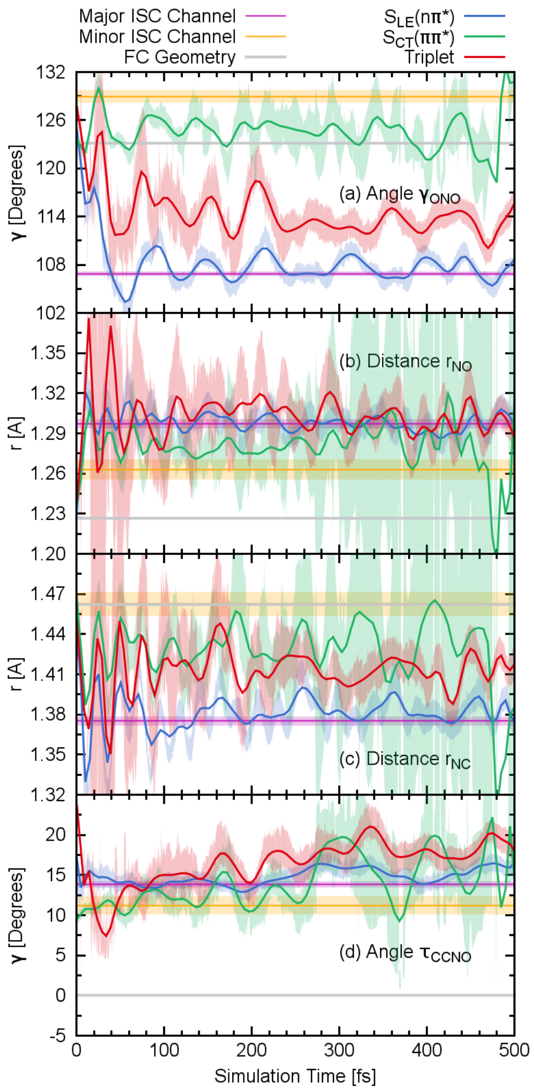


Figure S11: Time evolution of the averages and standard deviations of internal coordinates of the trajectories in $S_{LE}(n\pi^*)$ (blue), $S_{CT}(\pi\pi^*)$ (green) or any triplet state (red), averages and standard deviation of internal coordinates at the hopping geometries of the major (violet) and minor (orange) ISC pathways, and reference internal coordinates at the FC geometry (gray).

(6) Chong, D. P.; van Lenthe, E.; van Gisbergen, S.; Bearends, E. J. *J. Comput. Chem.* **2004**, *25*, 1030–1036.

(7) Baerends, E. J. et al. *ADF2016, SCM, Theoretical Chemistry, Vrije Universiteit, Amsterdam, The Netherlands, http://www.scm.com*, 2016.

(8) Atkins, A. J.; González, L. *J. Phys. Chem. Lett.* **2017**, *8*, 3840–3845.

(9) Sun, L.; Hase, W. L. *J. Chem. Phys.* **2010**, *133*, 044313.

(10) Zobel, J. P.; Heindl, M.; Nogueira, J. J.; González, L. *manuscript in preparation*, 2017.

(11) Kojima, M.; Nagakura, S. *Bull. Chem. Soc. Jpn.* **1966**, *39*, 1262–1269.

(12) Vogt, R. A.; Crespo-Hernández, C. E. *J. Phys. Chem. A* **2013**, *117*, 14100–14108.

(13) Barbatti, M.; Grannuci, G.; Persico, M.; Ruckenbauer, M.; Vazdar, M.; Eckert-Maksić, M.; Lischka, H. *J. Photochem. Photobiol. A* **2007**, *190*, 228–240.

(14) Granucci, G.; Persico, M.; Toniolo, A. *J. Chem. Phys.* **2001**, *114*, 10608–10615.

(15) Granucci, G.; Persico, M. *J. Chem. Phys,* **2007**, *126*, 134114.

(16) Granucci, G.; Persico, M.; Zoccante, A. *J. Chem. Phys.* **2010**, *133*, 134111.

(17) Plasser, F.; Ruckenbauer, M.; Mai, S.; Oppel, M.; Marquetand, P.; González, L. *J. Chem. Theory Comput.* **2016**, *12*, 1207–1219.

(18) Tapavicza, E.; Tavernelli, I.; Rothlisberger, U.; Filippi, C.; Casida, M. E. *J. Chem. Phys.* **2008**, *129*, 124108.

(19) Nangia, S.; Jasper, A. W.; Miller III, T. F.; Truhlar, D. G. *J. Chem. Phys.* **2004**, *120*, 3586–3597.

(20) Plasser, F.; Lischka, H. *J. Chem. Theory Comput.* **2012**, *8*, 2777–2789.

(21) Plasser, F.; Wormit, M.; Dreuw, A. *J. Chem. Phys.* **2014**, *141*, 024106.

(22) Mewes, S. A.; Mewes, J.-M.; Dreuw, A.; Plasser, F. *Phys. Chem. Chem. Phys.* **2016**, *18*, 2548–2563.

(23) Plasser, F. *TheoDORE 1.4: a package for theoretical density, orbital relaxation, and exciton analysis; available from* `http://theodore-qc.sourceforge.net`.

(24) El-Sayed, M. A. *Acc. Chem. Res.* **1968**, *1*, 8.

(25) Kurtz, K.; Hofmann, A.; de Vivie-Riedle, R. *J. Chem. Phys.* **2001**, *114*, 6151–6159.

(26) Plasser, F.; Barbatti, M.; Aquino, A. J. A.; Lischka, H. *J. Phys. Chem. A* **2009**, *113*, 8490–8499.

(27) Vogt, R. A.; Reichardt, C.; Crespo-Hernández, C. E. *J. Phys. Chem. A* **2013**, *117*, 6580.

(28) Crespo-Hernández, C. E.; Vogt, R. A.; Sealey, B. *Mod. Chem. Appl.* **2013**, *1*, 1000106.
